# Combined X‐Ray Emission Spectroscopy at Phosphorus and Nickel: Detecting Subtle Changes in Catalyst Electronic Structure at High Resolution

**DOI:** 10.1002/smll.202505199

**Published:** 2025-06-26

**Authors:** Zachary Mathe, Serena DeBeer

**Affiliations:** ^1^ Department of Inorganic Spectroscopy Max Planck Institute for Chemical Energy Conversion Stiftstr. 34–36 45470 Mülheim an der Ruhr Germany

**Keywords:** Ab initio calculations, homogeneous catalysis, phosphorus, valence‐to‐core X‐ray emission spectroscopy, X‐ray absorption spectroscopy

## Abstract

Valence‐to‐core X‐ray emission spectroscopy (VtC XES) is widely used to characterize valence electronic structure, especially of transition metal systems in homogeneous and bioinorganic catalysis. Although metal K‐edge VtC XES has proved useful, its observable information content is limited by the large lifetime broadening of the metal 1*s* core hole, and its practical application is limited by small VtC emission probability and thus low count rates. Ligand VtC XES in transition metal complexes, though largely unexplored, offers a higher resolution and potential for broad applications in catalysis research. Here, P VtC XES is introduced for catalysts with phosphine ligands, perhaps the most important class of spectator ligands in homogeneous catalysis. P VtC XES is sensitive to subtle changes in electronic structure, with difference spectra that are well‐reproduced by density functional theory (DFT) calculations, indicating that DFT can not only provide insight into the physical origins of spectral features but can also facilitate the identification of unknown species. Comparison to Ni VtC XES, as well as previously published X‐ray absorption data, establishes the high resolution and complementary benefits of the technique. The potential of P VtC XES as a metal‐ and spin‐agnostic tool for experimentally assessing electronic structure and mechanisms in phosphine‐coordinated catalysts is highlighted.

## Graphical abstract

Phosphorus valence‐to‐core X‐ray emission spectroscopy (VtC XES) is introduced as a highly sensitive, spin‐ and metal‐agnostic probe of valence electronic structure in bisphosphine catalysts. Calculations with density functional theory (DFT) accurately reproduce difference spectra of similar species and provide structure‐spectrum relationships. Combined P VtC XES and DFT is a promising program for the investigation of catalytic mechanisms.

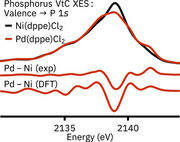

## 1 Introduction

Kβ X‐ray emission spectroscopy (XES), including valence‐to‐core (VtC) XES, is widely employed to characterize valence electronic structure, especially of transition metal systems.^[^ ^1^, ^2^ ^]^ In a molecular orbital (MO) framework, 3*d* metal VtC XES results from transitions from primarily ligand‐localized orbitals to a metal 1*s* hole, with dipole transition intensity derived from the mixing of a small amount of metal *p* character in the donor MOs. Interpreted together with density functional theory (DFT) calculations, VtC XES can offer a map of local valence electronic structure, scaled by metal‐ligand orbital mixing, that can be related to, e.g., ligand identity, protonation state, and bond activation in chemically familiar terms. VtC XES has been used, for example, to identify the interstitial carbide in the N_2_‐binding cofactor of nitrogenase,^[^ ^3^, ^4^ ^]^ assign intermediates in ferricyanide photochemistry,^[^ ^5^ ^]^ and describe speciation of binding sites in a Cu‐zeolite catalyst.^[^ ^6^ ^]^ Key to such studies is the accurate calculation of spectra from candidate computational models.

For catalysis in particular, an electronic structural model built from coupled experimental and computational X‐ray spectroscopy offers a compelling bridge between established chemical concepts and real reactivity. Recent time‐resolved Rh L‐edge X‐ray absorption spectroscopy (XAS) on CpRh(CO)_2_ has probed L─M donation and M─L back‐donation during C─H activation, motivating rational design principles,^[^ ^7^ ^]^ and subsequent theoretical work suggested resonant VtC XES could provide even greater insight.^[^ ^8^ ^]^ Such experiments conducted with “tender” X‐rays (the Rh L edge is at 3 keV) are particularly promising because they offer a sharper natural linewidth than those with hard X‐rays at 3*d* K‐edges (>5 keV). Although 3*d* metal VtC XES has proved very useful, its observable information content can be limited by the large lifetime broadening of the metal 1*s* core hole and its practical application is complicated by small emission probabilities, as well as the prevalence of transitions not apparently resulting from one‐electron, one‐photon transitions.^[^ ^9^, ^10^, ^11^ ^]^

Ligand VtC XES probes valence MOs at high resolution through strongly dipole‐allowed transitions. The relatively limited number of studies so far reflects the availability of tender XES instrumentation, which is recently increased.^[^ ^12^, ^13^, ^14^ ^]^ For example, oxidation of a Cu_4_S cluster resulted in larger changes in the sharper spectra for S VtC (2.5 keV) compared to Cu VtC (9.0 keV).^[^ ^15^ ^]^ Resonant VtC XES at sulfur has also probed covalency and *d*‐*d*‐like excited states in dithiolene complexes.^[^ ^16^ ^]^

Recently, we found P VtC XES of phosphate biomolecules to be highly sensitive to noncovalent interactions, especially hydrogen bonding, in both solid and solution samples.^[^ ^17^ ^]^ These results motivated the application of the technique to phosphines and specifically phosphine‐ligated coordination complexes. Phosphines are perhaps the most widely employed spectator ligands in organometallic homogeneous catalysis, with a great diversity of steric and electronic properties readily accessible^[^ ^18^, ^19^, ^20^ ^]^ (as well as important catalysts in their own right^[^ ^21^, ^22^ ^]^). A limited amount of experimental phosphine P VtC XES has been reported, with only a few older studies known to us.^[^ ^23^, ^24^ ^]^ A recent prospective computational survey of organophosphorus XAS and XES found the techniques to be more sensitive to different aspects of molecular structure, with XES perhaps more generally sensitive.^[^ ^25^ ^]^ P VtC XES promises similar information content to transition metal VtC XES for the same systems (i.e., a map of the valence electronic structure), at a higher linewidth and with highly dipole‐allowed transitions, thanks to the high P 3*p* character of the valence MOs.

Here, we demonstrate the sensitivity and utility of P VtC XES using a series of closely related divalent metal complexes: Ni(PPh_3_)_2_Cl_2_, Ni(PPh_3_)_2_Br_2_, Ni(dppe)Cl_2_, Ni(dppp)Cl_2_, and Pd(dppe)Cl_2_, as well as the free ligand PPh_3_. These five complexes have been variously employed and compared in catalysis, in particular for cross‐coupling and polymerization reactions, for decades.^[^ ^18^, ^26^, ^27^, ^28^ ^]^ For example, the highly active and industrially relevant Suzuki‐Miyaura catalysts Ni(dppe)Cl_2_ and Ni(dppp)Cl_2_,^[^ ^18^, ^29^, ^30^ ^]^ which differ only in the bisphosphine alkyl linker (CH_2_)_n = 2,3_, have quite different performance, and even different rate‐determining steps for polymerization reactions.^[^ ^31^, ^32^, ^33^, ^34^, ^35^ ^]^ Precatalysts of the form Ni(PPh_3_)_2_X_2_ have both historic and current importance for a variety of couplings.^[^ ^18^, ^26^ ^]^ In the present study, we first demonstrate the information content of Ni VtC XES for these complexes and then show the added information that can be obtained from P VtC XES. Calculations with density functional theory (DFT) allow the full assignment of spectra and an understanding of the geometric and electronic structural determinants of features. The relative strengths of XES versus XAS at both Ni and P are discussed. These studies highlight the potential of P VtC XES for elucidating mechanisms in homogenous catalysis.

## 2 Results and Discussion

Five bis‐phosphine complexes of *d* ^8^ divalent metals, as well as the free ligand PPh_3_, were chosen to illustrate the high information content and structural sensitivity of P VtC XES: Ni(PPh_3_)_2_Cl_2_, Ni(PPh_3_)_2_Br_2_, Ni(dppe)Cl_2_, Ni(dppp)Cl_2_, and Pd(dppe)Cl_2_; for the Ni complexes, Ni VtC XES was measured as well. Calculations were performed with TPSSh+X2C+CPCM/x2c‐TZVPall using ORCA 6.0,^[^ ^36^, ^37^, ^38^, ^39^, ^40^, ^41^, ^42^ ^]^ and all calculated structures are in reasonable agreement with published X‐ray crystallography.^[^ ^43^, ^44^, ^45^, ^46^, ^47^, ^48^, ^49^ ^]^ Some calculated properties are summarized in **Table** **1**. The three complexes of chelating bisphosphines are square‐planar and diamagnetic, while the bis(triphenylphosphine) complexes are tetrahedral triplets. All VtC transitions were calculated with DFT using two methods: a simple one‐electron‐one‐orbital method (1e1o), in which intensities are obtained from dipole matrix elements between canonical MOs,^[^ ^50^ ^]^ as well as time‐dependent DFT (TD‐DFT).^[^ ^51^ ^]^ Spectra were calculated from DFT transitions^[^ ^52^, ^53^ ^]^ using a Voigt profile with a relatively small Gaussian broadening to make all features clearly visible. See the Supporting Information for computational details and for further discussion of calculation methods and parameters.

**Table 1:** Calculated properties of the studied species.

Property^a)^	Ni(dppe)Cl_2_ ^b)^	Ni(dppp)Cl_2_ ^c)^	Ni(PPh_3_)_2_Cl_2_ ^d)^	Ni(PPh_3_)_2_Br_2_	Pd(dppe)Cl_2_	PPh_3_
Spin *S*	0	0	1	1	0	0
M─P (Å)	2.132	2.152	2.278	2.287	2.240	
M–X (Å)	2.214	2.213	2.234	2.367	2.391	
P─C (Å)	1.819	1.823	1.817	1.815	1.818	1.834
P–M–P (°)	86.7	90.4	106.3	105.8	85.6	
P–M–X (°)	95.4	92.1	127.4	126.6	93.4	
X–M–X (°)	132.0	131.9	105.4	105.7	133.2	
τ_4_ at Ni^e)^	0.07	0.07	0.90	0.87	0.06	
τ_4_ at P^f)^	0.93	0.89	0.91	0.91	0.93	0.90
P 1*s* (eV)	−2099.86	−2099.85	−2099.69	−2099.71	−2100.11	−2098.65
Ni 1*s* (eV)	−8236.08	−8236.09	−8237.35	−8237.41		
P Charge^g)^	+0.231	+0.229	+0.213	+0.210	+0.174	+0.058
M Charge	−0.109	−0.106	+0.030	+0.018	+0.430	
X Charge	−0.397	−0.381	−0.375	−0.371	−0.557	

^a)^ M = Ni or Pd; X = Cl or Br;

^b)^ dppe = (ethane‐1,2‐diyl)bis(diphenylphosphane);

^c)^ dppp = (propane‐1,3‐diyl)bis(diphenylphosphane);

^d)^ PPh_3_ = triphenylphosphine;

^e)^ τ_4_ parameter from Yang et al.,^[^ ^54^ ^]^ which characterizes a 4‐coordinate geometry on a scale of zero (square‐planar) to one (tetrahedral);

^f)^ For PPh_3_, the fourth vector for τ_4_ passes through the centroid of the phenylic carbons;

^g)^Charges calculated by Hirshfeld's method.^[^ ^55^ ^]^

### 2.1 Ni VtC XES

**Figure 1:**
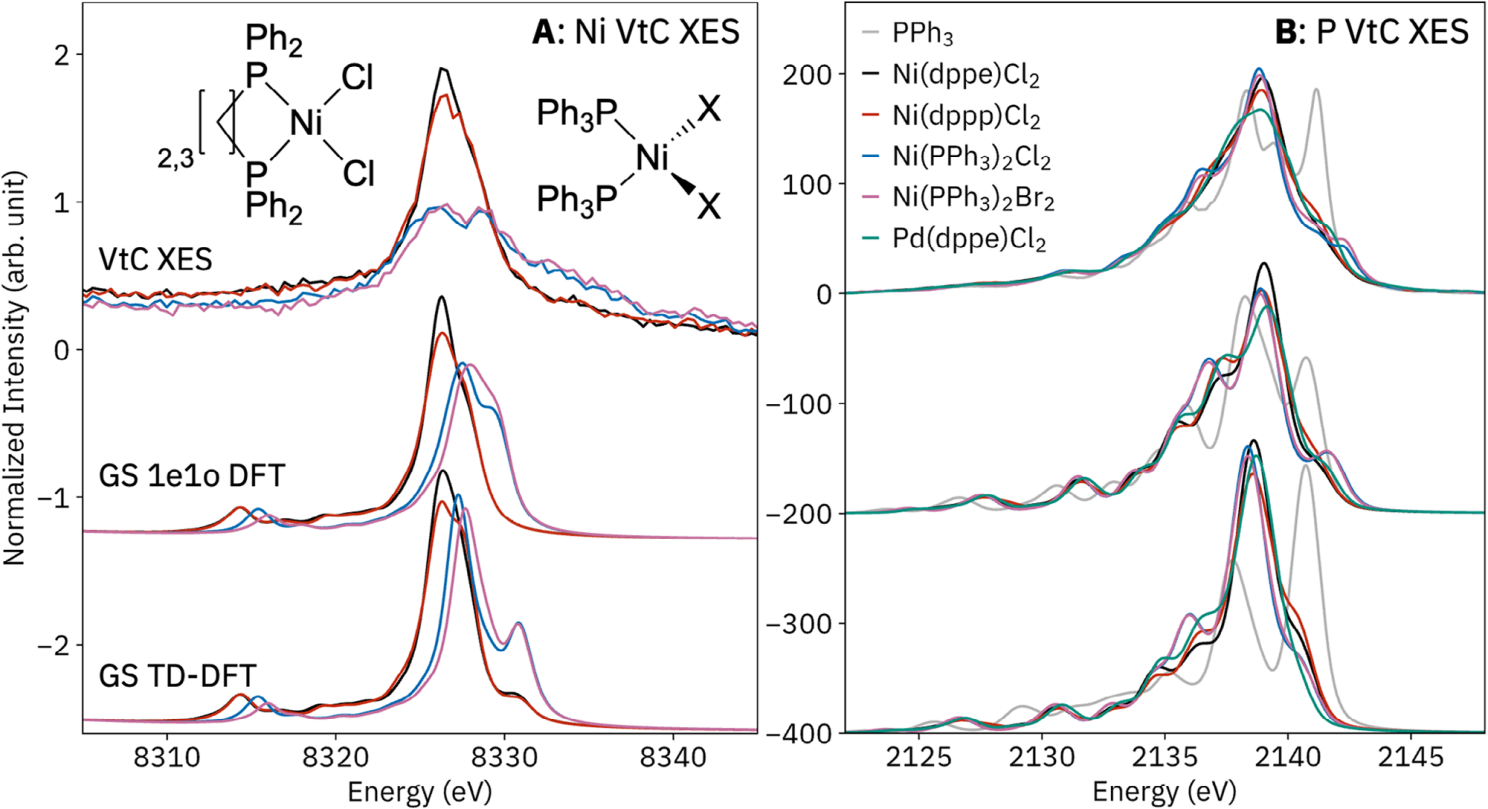
Valence‐to‐core X‐ray emission spectra (VtC XES) at the Ni K‐edge (left) and P K‐edge (right), including calculations below using a ground‐state one‐electron orbital difference method (GS 1e1o) as well as time‐dependent DFT (TD‐DFT). Structures of Ni(dppe)Cl_2_/Ni(dppp)Cl_2_ (left) and Ni(PPh_3_)_2_Cl_2_/Ni(PPh_3_)_2_Br_2_ (right) are shown inset.

Before introducing phosphine P VtC XES, we present the well‐established metal VtC XES for the Ni complexes (**Figure** **1**A). The Ni VtC XES is similar for each geometry: in the tetrahedral complexes, *p*‐*d* mixing is allowed, resulting in dipole intensity for formal 3*d→*1*s* transitions on the high‐energy side of the Ni VtC region.^[^ ^11^ ^]^ The long tails for the tetrahedral complexes continue beyond the HOMO‐LUMO gap (≈8333 eV), likely resulting from emission from multiply ionized intermediate states.^[^ ^9^, ^10^, ^56^, ^57^, ^58^ ^]^ Some differences are also clear within each geometry. The intensity of the single Kβ_2,5_ peak is lower for Ni(dppp)Cl_2_ versus Ni(dppe)Cl_2_, reflecting the lower M─L covalency and thus Ni *p* character in the ligand MOs. The entire Kβ_2,5_ region is shifted to higher energy for Ni(PPh_3_)_2_Br_2_ versus Ni(PPh_3_)_2_Cl_2_, a result of the higher‐energy valence *p* orbitals of bromide versus chloride.^[^ ^9^, ^59^ ^]^ Both of these differences, discussed further below together with the P VtC XES, are reproduced by both DFT methods. The inclusion of quadrupole transitions, as well as their method of calculation, affected some spectra; see Figure S4 (Supporting Information) for further discussion.

The Ni Kβ mainlines (3*p*→1*s*) are very similar for each geometry: the diamagnetic complexes have a single Kβ_1,3_ feature, while the high‐spin bis(triphenylphosphine) complexes additionally have a Kβ′ shoulder resulting from 3*p*‐3*d* exchange in the 3*p* ^5^3*d* ^2^ final states (Figure S1, Supporting Information). Calculation of Ni Kβ mainlines would require proper treatment of the 3*p* ^5^3*d* ^8^ multiplet problem, which requires a wavefunction‐based approach that is beyond the scope of the present study.^[^ ^60^ ^]^

### 2.2 P VtC XES: Orbital Structure

**Figure 2:**
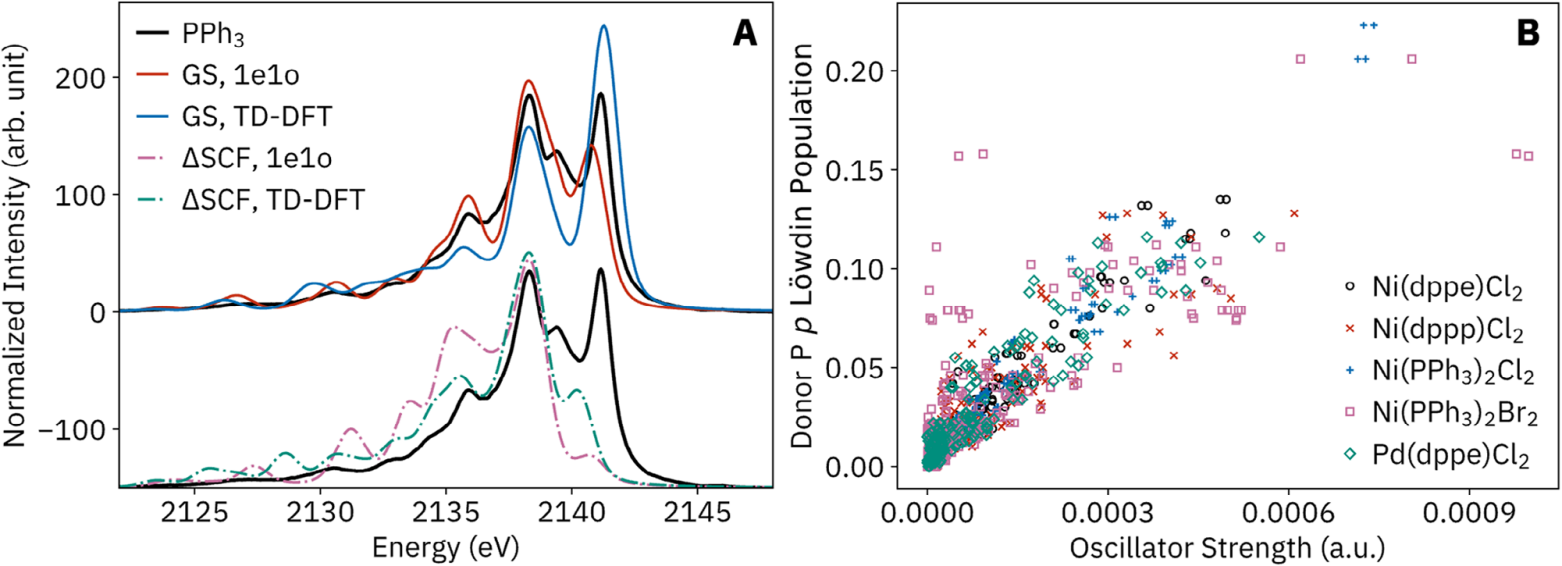
A) Experimental P VtC XES of PPh_3_ (black) with spectra calculated by four methods (colored): 1‐electron‐1‐orbital (1e1o) and TD‐DFT calculations, with either a ground‐state (GS) or core‐hole ΔSCF wavefunction reference, all shifted in energy to align the peaks at 2138 eV. The root mean square deviations compared to the experiment for the entire plotted range were 14 (GS, 1e1o), 23 (GS, TD‐DFT), 41 (ΔSCF, 1e1o), and 31 (ΔSCF, TD‐DFT). B) relation between P VtC XES oscillator strength versus donor MO Löwdin P *p* population.

All P VtC XES spectra are presented together in Figure 1B to show the total breadth of variation. Before proceeding to the coordination complexes, we analyze PPh_3_ to assess different calculation methods and illustrate general electronic structural features of organophosphines and their P VtC spectra. Spectra were calculated by either the 1e1o or TD‐DFT method, using either a ground‐state (GS) or core‐hole ΔSCF wavefunction (**Figure** **2**A).^[^ ^51^, ^61^, ^62^ ^]^ Both GS methods approximately captured the energies of the most prominent peaks at 2136, 2138, and 2141 eV, and the most accurate intensities for these peaks and the lowest overall root mean square deviation (RMSD) from the experiment were obtained by the GS 1e1o method. The GS 1e1o approach is also the most user‐friendly and computationally inexpensive and has previously been found to be effective for phosphate P VtC XES;^[^ ^17^, ^63^ ^]^ thus, all following spectra are presented with this method. All four methods overestimated the intensities of lower‐energy transitions, which is also a typical shortcoming of transition metal VtC XES calculations.^[^ ^9^, ^51^ ^]^ All P VtC XES transitions were purely dipole‐allowed, and across all systems, the P *p* character in the donor MO was found to be necessary for high transition intensity, but not the sole factor (Figure 2B), similar to the results of previous metal VtC XES calculations.^[^ ^9^, ^64^ ^]^

**Figure 3:**
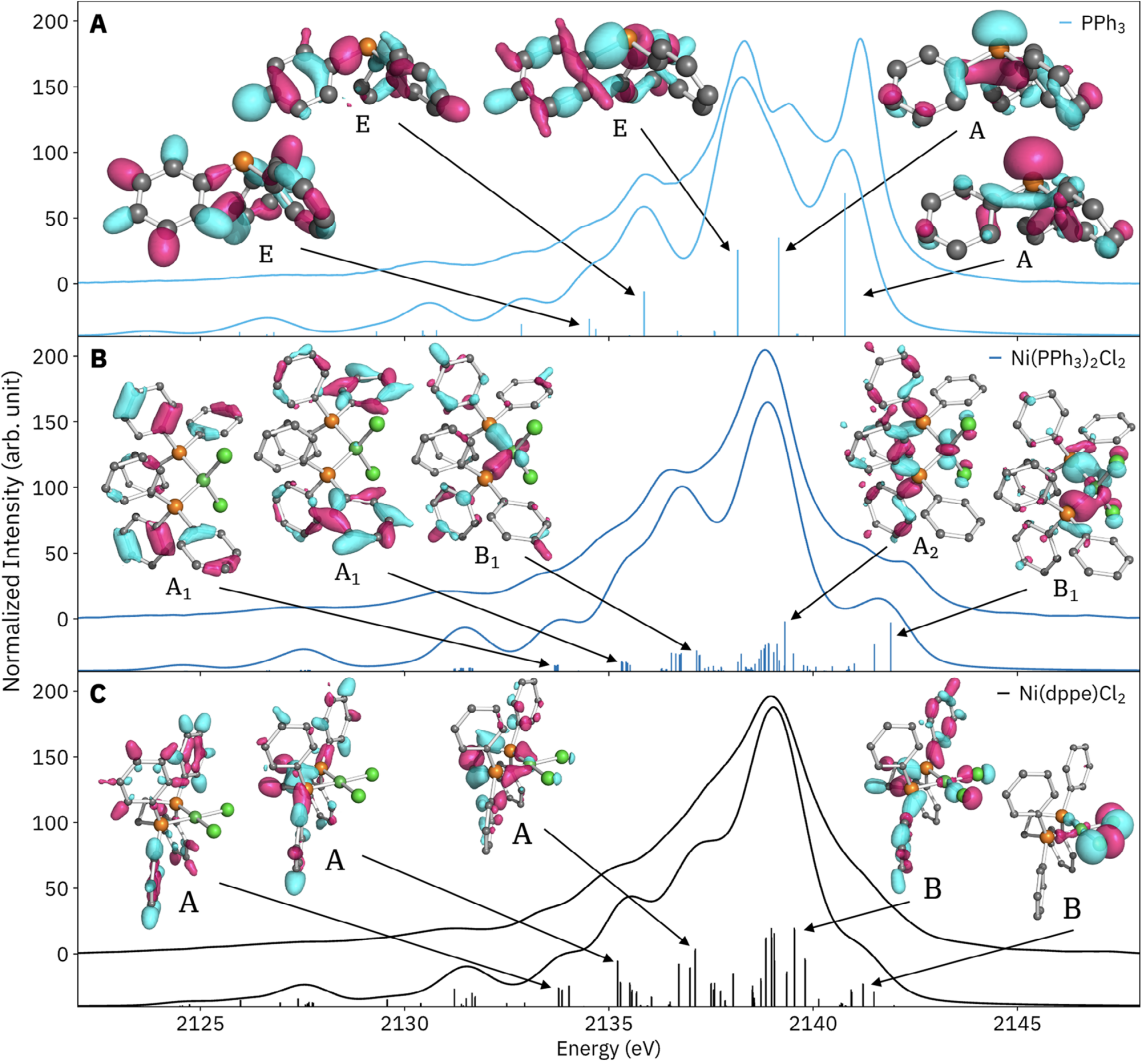
Experimental (top) and calculated (bottom) P VtC XES of PPh_3_ (A, C_3_), Ni(PPh_3_)_2_Cl_2_ (B, C_2v_), and Ni(dppe)Cl_2_ (C, C_2_) with some significant donor orbitals plotted and labeled according to their approximate symmetry.

PPh_3_ has C_3_ symmetry, resulting in MOs of *A* and *E* symmetry, both of which have dipole‐allowed transitions to the *A* P 1*s* core hole; thus, symmetry alone does not allow the MO assignment spectral features. The character of transitions calculated by the GS 1e1o method are assessed by plotting the donor MOs (**Figure** **3**).^[^ ^17^, ^50^, ^59^ ^]^ The two highest‐energy features each result from one *A* donor MO, the HOMO and HOMO‐1, which have primarily P lone‐pair character and some P─C π and phenyl π character. The central feature at 2138 eV, as well as its lower‐energy shoulders, primarily results from *E* orbitals with P─C σ and phenyl σ character. As was demonstrated for Ca VtC XES of large systems,^[^ ^59^ ^]^ the spatial extent of donor MOs indicates which (electronic‐)structural features *can*, but not necessarily *do*, influence spectral features. Intense transitions can result from MOs with majority density on distal regions of the structure, the omission of which does not significantly affect the calculated spectrum, because canonical MOs are maximally delocalized. See Figures S5 and S6 (Supporting Information) for localized orbitals, overlaps between localized and canonical MOs, and further discussion. Overall, care must be taken in deducing structure‐spectrum relationships from inspection of calculated MOs.

Alkyl and phenyl phosphines are strong σ donors, which is reflected in the P VtC XES of the complexes versus free PPh_3_: the largest change is the loss of the isolated P lone‐pair feature. This difference could be particularly helpful in the deconvolution of reaction mixtures with an excess of free phosphine ligand, a common requirement for efficient catalysis.^[^ ^65^, ^66^, ^67^, ^68^ ^]^ Upon binding, P 1*s* orbitals are stabilized by at least 1 eV, a result of the dative (charge‐transfer) character of the P─M^2+^ bond (Table 1). The dominant spectral features are more similar within the square‐planar and tetrahedral sets of complexes; calculated spectra with representative donor MOs for Ni(dppe)Cl_2_ and Ni(PPh_3_)_2_Cl_2_ are plotted in Figure 3B,C. For both species, Ni 3*d* and Cl 3*p* characters are found for the main peak and higher‐energy shoulders, while P─C σ and phenyl σ characters are found for all but the highest‐energy transitions. The intense transitions underlying the main peaks all have significant P─C σ bonding character. For Ni(PPh_3_)_2_Cl_2_, the highest‐energy transitions are about +0.8 eV higher in energy and have more Ni 3*d* character. A notable Ni─P σ bonding character is found in the first low‐energy shoulder, ≈2136–2137 eV, for both species.

### 2.3 P VtC XES: Determining Factors

**Figure 4:**
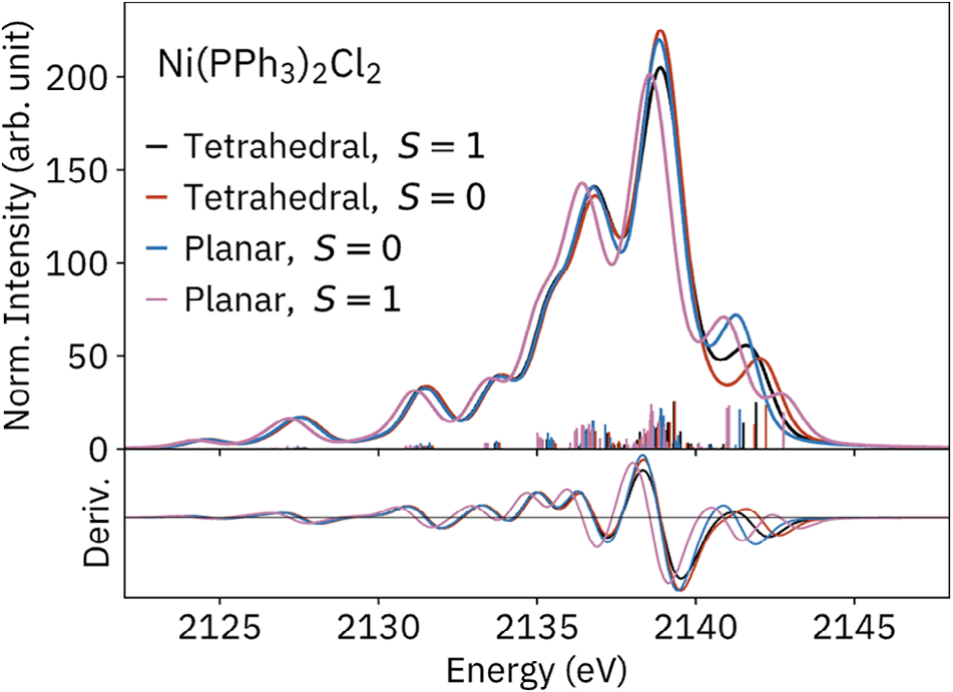
Calculated P VtC XES for Ni(PPh_3_)_2_Cl_2_ using S = 0,1 and either a tetrahedral (optimized with S = 1) or trans‐square‐planar (optimized with S = 0) geometry, with derivatives below.

Next, calculations using hypothetical geometries and wavefunctions are used to explore the geometric and electronic factors that influence the shape of P VtC XES spectra. Owing to the intermediate net ligand field of chloride and phosphine, the typically tetrahedral Ni(PPh_3_)_2_Cl_2_ can adopt a diamagnetic, *trans*‐square‐planar conformation in certain conditions, e.g. co‐crystallization with CH_2_Cl_2_.^[^ ^43^, ^69^ ^]^ The effects of geometry and spin state on the P VtC XES of Ni(PPh_3_)_2_Cl_2_, and thus the prospects of distinguishing such variations in an unknown solution, were investigated with DFT (**Figure** **4**). The spectra are dependent on both geometry and spin state, with the spin state more strongly modulating the main peak intensity and both parameters strongly effecting the intensity and position of the high‐energy shoulders that are most sensitive to Ni 3*d* character. With a high‐spin wavefunction and square‐planar geometry (Figure 4, pink trace), the entire spectrum is shifted to lower energy, a result of P 1*s* destabilization by ≈0.4 eV upon half‐occupation of the 3*d*_x2‐y2_ orbital (which has lobes directed at the ligands). These results indicate that P VtC XES, as well as metal Kβ XES, could be used to investigate variable spin states of active catalysts, which can be an important factor governing reaction mechanisms and selectivity, as well as systems with complex electronic structures.^[^ ^70^, ^71^, ^72^, ^73^, ^74^ ^]^

**Figure 5:**
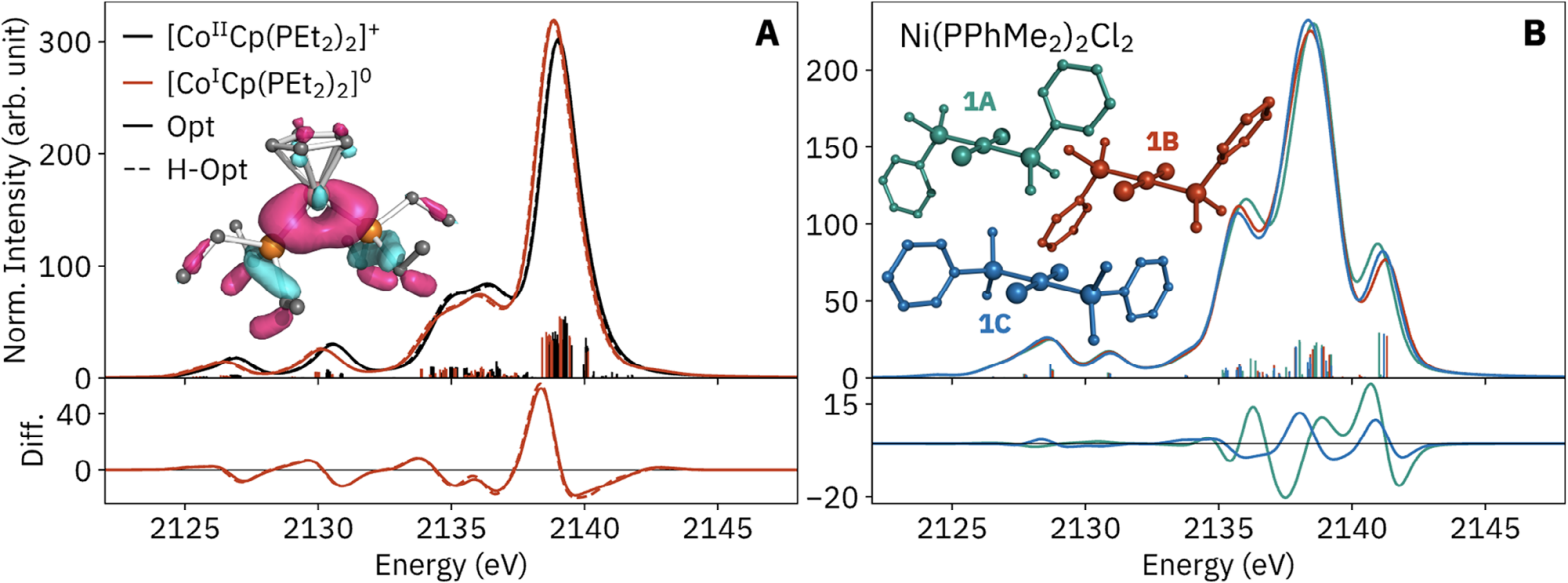
A) Calculated P VtC XES for [Co^II^Cp(PEt_3_)_2_]^+^ and [Co^I^Cp(PEt_3_)_2_]^0^, with differences (Co^I^ – Co^II^) below; equivalent spectra are obtained using crystallographic coordinates with only H optimized (H‐Opt)^[^ ^80^ ^]^ and with fully optimized structures (Opt). The donor MO for the strongest transition of [Co^I^Cp(PEt_3_)_2_]^0^ is plotted inset. B) Calculated P VtC XES for Ni(PPhMe_2_)_2_Cl_2_ in three conformations: with the Ni─P─C(Ph) planes normal (**1A**, green, and **1B**, red) or coincident (**1C**, blue) to the Ni coordination plane, and with the phenyl groups coincident (**1A**, green) or normal (**1B**, red) with the Ni─P─C(Ph) planes. Difference spectra are plotted below (**1A** – **1B**, green; **1C** – **1B**, blue). All structures have an inversion center at Ni and are shown with the Ni coordination plane in the same orientation.

In addition to being strong σ donors, phosphines, especially when bound to lower‐valent metal centers, are π acceptors. M─P back‐bonding is typically described as a donation into P─C σ^*^ orbitals and correlates with the lengthening of P─C bonds.^[^ ^75^, ^76^, ^77^, ^78^, ^79^ ^]^ For example, an X‐ray crystallography study found that reduction of [Co^II/I^Cp(PEt_3_)_2_]^+/0^ lengthened P─C distances by +0.017 Å and shortened Co─P distances by ‐0.012 Å, more than canceling the typical effect of *d*‐orbital and M─L expansion upon metal reduction.^[^ ^80^ ^]^ Calculated P VtC spectra of these complexes (**Figure** **5**A) show dramatic effects of reduction: the entire spectrum shifts to lower energy, the main peak increases in intensity, and the lower‐energy shoulders decrease in intensity. The energy shift of ≈−0.2 eV reflects the greater destabilization of the P 1*s* orbitals (+0.8 eV) than the relevant valence MOs (+0.6 eV within 2138.4–2149.5 eV) by the additional 3*d* electron. The most intense transitions result from MOs with significant P─Co σ character, which have increased P *p* participation and thus spectral intensity when the P─Co distance is shorter. The higher‐lying 3*d*_yz_ SOMO/HOMO does not directly contribute significantly to the spectra, but its occupation strongly perturbs the lower‐energy valence electronic structure probed by P VtC XES.

The large π‐mediated spectral changes with metal reduction suggest that P VtC XES could also be sensitive to conformation, in particular, the orientations of P─C bonds and ligand π systems relative to occupied 3*d* orbitals. This hypothesis was explored computationally using Ni(PPhMe_2_)_2_Cl_2_, a selective Suzuki‐Miyaura coupling catalyst,^[^ ^81^ ^]^ chosen for its conformational flexibility. Three constraint‐optimized structures and their spectra are shown in Figure 5B: two structures with the Ni─P─C(Ph) planes normal to the Ni coordination plane that differ by the rotation of the phenyl groups (**1A** and **1B**), and one structure with the Ni─P─C(Ph) plane coincident with the Ni coordination plane (**1C**). Rotation of the P‐phenyl bonds has a large impact on the P VtC spectrum (**1B** vs **1A**, green difference) despite the enthalpy difference of the structures being only 0.08 kcal mol^−1^. Although the rotation of the P─Ni bonds relative to the Ni coordination plane results in a larger change in enthalpy (4.5 kcal mol^−1^; **1B** vs **1C**, blue difference), the effect on the P VtC spectrum is smaller. Differences in back bonding were not readily apparent in the calculations: Ni─P and P─C distance all increased by ≤0.003 Å, and natural orbitals for chemical valence (NOCV) fragment analysis did not reveal significant Ni─P π interactions. The spectral differences may instead be explained by the orientation of the phenyl π system versus the Ni 3*d* orbitals; see Figure S7 (Supporting Information). In summary, these calculations show that P VtC XES could be highly sensitive to subtle electronic structural changes related to ligand and substrate conformation, as well as metal charge and spin state.

### 2.4 P VtC XES: Chemical Sensitivity

Having understood the key relationships between P VtC XES and electronic and geometric structure, we are now prepared for an informed analysis of the experimental and calculated difference spectra for pairs of complexes. The systematic characterization of bisphosphine ligation properties and efficacy for catalysis, especially bound to Ni and Pd, has garnered interest for decades.^[^ ^18^, ^82^, ^83^, ^84^ ^]^ Along with bite angle,^[^ ^82^ ^]^ parameterizations of steric bulk have long dominated, from Tolman's cone angle^[^ ^85^ ^]^ in the 1970s to modern tools, such as those using percent buried volumes,^[^ ^19^ ^]^ that better predict denticity and reactivity. Although sterics can accurately predict many resting‐state structures, electronics are also important for selectivity and actually achieving the steps of catalysis, and the two effects may not be neatly separable.^[^ ^19^, ^20^, ^82^, ^83^, ^86^, ^87^ ^]^ Here, we have explored the chemical sensitivity of P VtC XES using well‐characterized, isolable systems, in order to demonstrate its potential for investigating more complex systems. Overall, the calculated difference spectra accurately capture the main differences between highly similar species (albeit with typically exaggerated intensities).

**Figure 6:**
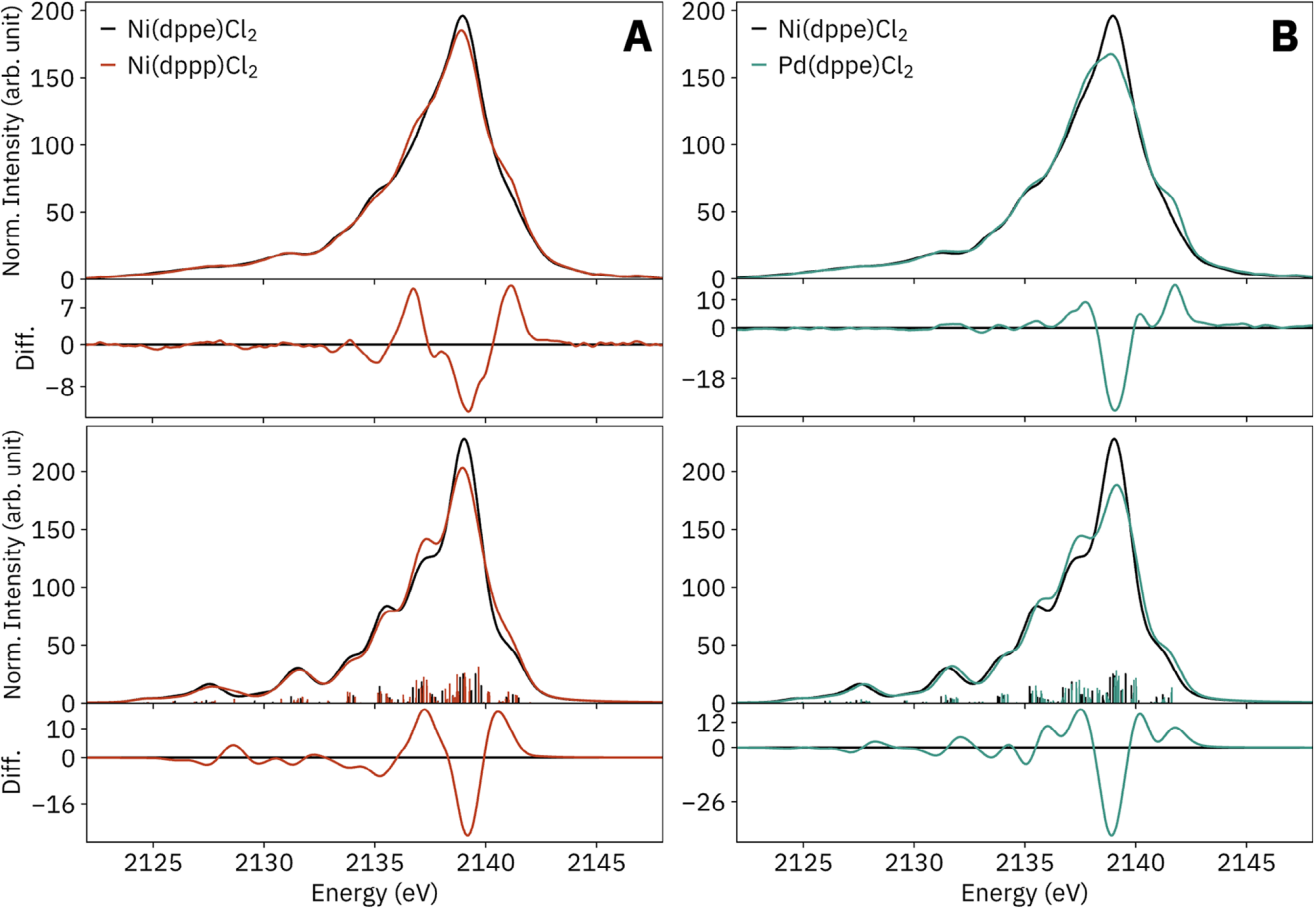
Experimental (top) and calculated (bottom) P VtC XES difference spectra of Ni(dppp)Cl_2_ A) and Pd(dppe)Cl_2_ B) versus Ni(dppe)Cl_2_.

P XES VtC difference spectra of Ni(dppp)Cl_2_ and Pd(dppe)Cl_2_ versus Ni(dppe)Cl_2_ are provided in **Figure** **6**, showing the effects of alkyl linker and metal variation. Compared to that of Ni(dppe)Cl_2_, the spectrum of Ni(dppp)Cl_2_ has decreased intensity of the main peak, increased intensity of the highest‐intensity shoulders to both higher and lower energy (≈2137 and 2141 eV), and reduced intensity of the lowest‐energy shoulders (≈2135 eV), all well‐reproduced by DFT. These differences may result from the longer alkyl linker itself, as well as differing orbital interactions with the [NiCl_2_] moiety.^[^ ^20^, ^82^, ^88^, ^89^ ^]^ Compared to Ni(dppe)Cl_2_, Ni(dppp)Cl_2_ has lower P─Ni covalency, as evidenced by longer Ni─P distances (+0.020 Å) and a lower Mayer bond order (−0.04). Lower M─P covalency for M(dppp)Cl_2_ has also been supported by P and Pd XAS.^[^ ^90^ ^]^ Ni(dppp)Cl_2_ also has a slightly larger bite angle (+3.8°) (Table 1) and different phenyl orientations relative to the Ni coordination plane (Figure S15, Supporting Information), both of which affect P orbital mixing (Figure 5).^[^ ^91^ ^]^ Additionally, the longer alkyl linker itself is more electron‐rich, leading to a slightly higher P lone‐pair occupancy for dppp (1.90 vs 1.89, on a scale of 1.81–1.94 for Sigman's bisphosphines library).^[^ ^83^ ^]^

To investigate the alkyl versus chelation effects, calculations were performed on a variety of structure pairs that emphasize each effect (Figure S16, Supporting Information): frozen and reoptimized free ligands (alkyl effect), free PPh_2_(CH_2_)_n = 1,2_CH_3_ ligands (alkyl effect), and frozen complexes with the alkyl linkers replaced with hydride or methyl groups (chelation effect). In the models that probe chelation effects, both the increase of the first low‐energy shoulder at 2137 eV and the decrease of the lower‐energy shoulder at 2135 eV, observed in the experiment and the full calculation, are retained. Because the 2137‐eV feature corresponds to a significant P─Ni σ bonding character (Figure 3), it is reasonable to expect it to be most sensitive to coordination geometry. However, the difference in relative shoulder intensities is also conserved in the alkyl effect calculations, a result that might be surprising if the wide distribution of P─C σ character (Figure 3) were not considered. Thus, the differences between Ni(dppp)Cl_2_ and Ni(dppe)Cl_2_ P VtC XES cannot be neatly binned into separate alkyl and chelation effects. These observations demonstrate the care that must be taken in deducing structure‐spectral relationships from simple MO assignments and the value of interpreting full calculations, with the help of hypothetical models, to understand spectra.

Ni and Pd phosphine catalysts have been compared extensively; while Pd‐based catalysis often proceeds with lower temperature and catalyst loading, performance improvement with the more abundant Ni is an active area of research.^[^ ^18^, ^30^, ^84^, ^92^ ^]^ P VtC XES offers a metal‐agnostic probe of valence electronic structure that could help understand the differences in catalysis between similar systems. In Figure 6B, Pd(dppe)Cl_2_ has a much less intense main peak and more prominent shoulders compared to Ni(dppe)Cl_2_, and again, DFT provides an accurate difference spectrum (Figure 6B). Compared to Ni(dppe)Cl_2_, Pd(dppe)Cl_2_ has longer metal bond distances (M─P +0.108 Å, M─Cl +0.176 Å) and a slightly smaller bite angle (−1.1°), thanks to the larger size of Pd^2+^ (Table 1), while the P─C distances are nearly identical, suggesting there is no significant difference in M→P back‐bonding. Analysis of the electronic structures shows greater electron localization in P─M bond for the Pd complex: the electron density at the bond critical point is 7% higher and the P Hirshfeld charge is lower (−0.06), while the P sites have more stable 1*s* orbitals (−0.25 eV), implying a lower electron density very close to the P nuclei. This 1*s* stabilization causes the spectral shift en bloc to higher energy, while the higher P─M covalency increases the intensity of the high‐energy transitions with metal *d* character. This description is compatible with reported P XAS, in which the first feature, assigned as a 1*s→*P–M σ^*^ transition, was found to be more intense and +0.5 eV higher in energy in the Pd complex, changes attributed to higher M─P covalency (vide infra).^[^ ^90^ ^]^

**Figure 7:**
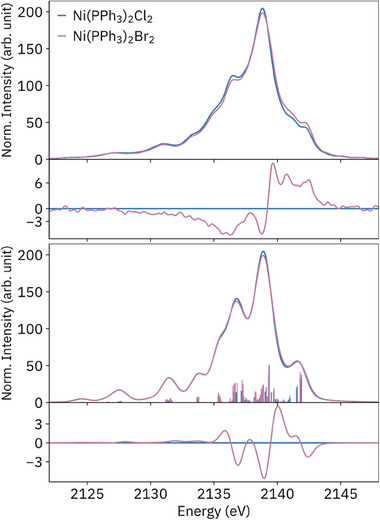
Experimental (top) and calculated (bottom) P VtC XES of Ni(PPh_3_)_2_X_2_, with difference spectra (bromide – chloride).

Although the chloride and bromide complexes have very similar structures (Table 1), their P VtC spectra are subtly but clearly differentiable: intensity is redistributed from the main peak and low‐energy shoulders to higher energy, which is reasonably reproduced by DFT (**Figure** **7**), although the number of high‐energy shoulder features is not correct in the calculated spectra. A ^1^H NMR study of spin delocalization onto the phenyls of Ni(PPh_3_)_2_X_2_ found that stronger halide π donation to Ni was responsible for the lower lability of the bromide complex as well as increased Ni→P π back‐bonding.^[^ ^93^ ^]^ Such increased Ni─P π mixing may be the cause of the higher intensity of the high‐energy shoulders in the bromide complex, which have significant Ni 3*d* character. However, stronger Ni─P (back‐)bonding is not evident from the calculated structures, as the M─P bond is slightly longer in the bromide complex. While in the P VtC, the high‐energy transitions differ in intensity, in the Ni VtC they differed in energy, intuitively reflecting the higher Br valence *p* orbital energies. These spectra demonstrate that P VtC XES is sensitive to quite small changes in metal coordination; the substitution of one chloride for a methyl or phenyl, for example, is calculated to result in much larger changes, with the *trans* P site most affected (Figure S17, Supporting Information).

### 2.5 P VtC XES and Other Techniques

**Figure 8:**
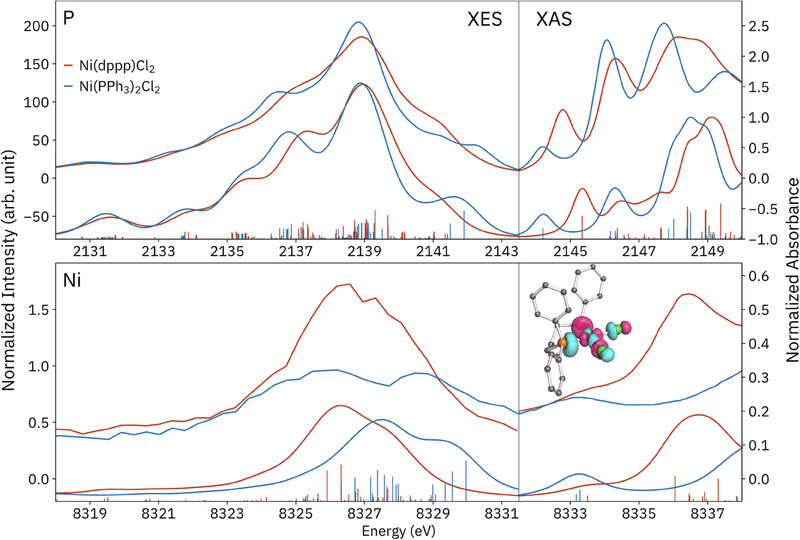
X‐ray emission (left) and absorption (right) spectra at the P (top) and Ni (bottom) K‐edges of Ni(dppp)Cl_2_ and Ni(PPh_3_)_2_Cl_2_, all plotted with the same energy scale. Calculations are shown below each spectrum, using the GS 1e1o method for XES with a +98.3‐eV shift for Ni, and GS TD‐DFT for XAS, with energy shifts of +48.0 and +102 eV for P and Ni, respectively. (We note that a prior report of TD‐DFT calculations of these spectra used different energy shifts for each species.^[^ ^90^ ^]^) The relative alignment of the P and Ni energy scales is approximate and was performed manually. A representative acceptor natural transition orbital (NTO) for the first Ni excited state of Ni(dppp)Cl_2_ is shown inset, demonstrating Ni 3*d*_x2‐y2_ and Ni─P σ^*^ character; the first acceptor NTO for the P K‐edge is nearly identical. P XAS spectra are adapted with permission from Donahue et al. 2015; copyright 2015 American Chemical Society.^[^ ^90^ ^]^ The Ni(dppp)Cl_2_ Ni XAS spectrum is adapted with permission from Saini et al. 2023; copyright 2023 Wiley‐VCH GmbH.^[^ ^57^ ^]^ The Ni(PPh_3_)_2_Cl_2_ Ni XAS spectrum is adapted with permission from Soma et al. 2016; copyright 2016 SBIC.^[^ ^58^ ^]^

Phosphine coordination complexes, including those presented here, have been subject to many spectroscopic techniques, including metal K‐ and L‐edge XAS,^[^ ^57^, ^90^, ^91^, ^94^ ^]^ metal Kβ mainline and VtC XES,^[^ ^95^ ^]^ P (and other ligand) K‐edge XAS,^[^ ^90^, ^94^ ^]^ and ^31^P NMR spectroscopies.^[^ ^32^ ^]^ Using Ni(dppp)Cl_2_ and Ni(PPh_3_)_2_Cl_2_, we evaluate the XES, together with previously‐reported XAS,^[^ ^57^, ^58^, ^90^ ^]^ at the P and Ni K‐edges, both in terms of the information content and availability of accurate calculations (**Figure** **8**). The clearest electronic structural difference between these species is the lower M─L covalency in the tetrahedral Ni(PPh_3_)_2_Cl_2_ that results from less favorable ligand‐metal σ orbital interactions, as evidenced by, e.g., longer bonds and XAS studies.^[^ ^90^, ^96^ ^]^

P and Ni VtC XES probe the same valence orbital manifold, and when the core and valence holes are neglected in the intermediate and final states, as in the present GS 1e1o method, these spectra are understood as deriving from the very same MOs, with intensities proportional to the square of the dipole matrix element with the Ni and P 1*s* orbitals, respectively. The most apparent experimental difference between P and Ni VtC XES is the 1*s* natural linewidth, 0.47 eV for P and 1.39 eV for Ni, which results in much broader spectra at Ni.^[^ ^97^ ^]^ Like the VtC spectra, the P and Ni K‐edge absorption spectra also probe similar states and are presented together with TD‐DFT calculations in Figure 8, which somewhat account for the presence of the core‐hole and promoted electron. Again, the improved resolution at the P K‐edge is clear.

The information content of P XAS for phosphine coordination complexes has been investigated in detail by the Daly group.^[^ ^90^, ^94^, ^98^ ^]^ The first two peaks, assigned to transitions to M─P σ^*^ and P─C π^*^ states respectively, have been interpreted in terms of parametrized metal‐ligand covalency, following prior work on ligand XAS.^[^ ^96^ ^]^ Higher‐energy features have not received much interpretation. In Figure 8, for both species and K edges, the first transitions have Ni─P σ^*^ (i.e., phosphine lone‐pair and Ni 3*d*_x2‐y2_) character.^[^ ^90^, ^99^ ^]^ At the P K‐edge, this feature is ≈0.6 eV higher in energy for Ni(dppp)Cl_2_ than for Ni(PPh_3_)_2_Cl_2_, which is to be expected: starting from P(lone‐pair) and Ni 3*d*_x2‐y2_ orbitals of similar energies, stronger mixing in the square‐planar complex results in a lower‐energy occupied Ni─P σ orbital and a higher‐energy unoccupied Ni─P σ^*^ orbital.^[^ ^90^, ^96^ ^]^ This effect might be thought to be mirrored in Figure 8 across the XES/XAS divide (which can be thought of roughly as the Fermi level or HOMO‐LUMO gap) in the VtC spectra, in which the last emission feature is found at lower energy for Ni(dppp)Cl_2_. However, inspection of the occupied MOs (Figure 8), which are generally highly delocalized, indicates much Ni─P σ character lies on the lower‐energy shoulders for both species, which have similar energies. Overall, the P XES and XAS provide high‐resolution, complementary information regarding the ground‐state electronic structure as well as low‐lying excited states that may play a role in reactivity.

As demonstrated here, accurate calculations are crucial to obtain insight from VtC XES of even medium‐sized molecules due to the density and complexity of transitions involved. More accurate spectra are obtained at P than at Ni for VtC XES, in terms of the relative energies of the most intense features between species, which indicates that the quality of the ground‐state DFT wavefunctions is likely not the problem at Ni. The agreement of relative transition energies with experiment is also generally better for the P XES versus P XAS; although TD‐DFT has provided important insights for P XAS, the qualitative agreement with the first few features is often, though not always, somewhat lacking.^[^ ^58^, ^90^, ^98^ ^]^ In particular, the error of 0.6 eV for the relative energies of the first features of Ni(PPh_3_)_2_Cl_2_ and Ni(dppp)Cl_2_, also reported by others,^[^ ^90^ ^]^ is quite large. The difference in quality for the different calculations could result from the importance of core‐hole effects in the intermediate or final states, generation of multiply ionized intermediate states, or shake processes. Determining the relative importance of these phenomena should guide future efforts to improve Ni (and 3*d* generally) VtC calculations.

Although not reported for the present complexes, Ni L‐edge XAS can also offer rich electronic information and a narrow linewidth and was recently applied to Ni‐2,2′‐bipyridine catalysts.^[^ ^74^ ^]^ However, 3*d* L‐edges require wavefunction‐based methods to simulate,^[^ ^100^ ^]^ which are much more demanding in both computational resources and user input compared to DFT, which can be readily applied to a vast library of structures.^[^ ^19^, ^25^, ^83^ ^]^ Other tools, such as ^31^P NMR and X‐ray crystallography, are more practical than X‐ray spectroscopy for assessing isolable systems with straightforward, diamagnetic electronic structures. ^31^P NMR spectroscopy has also been used to decipher mixtures and catalytic mechanisms of phosphine‐coordinated catalysts,^[^ ^68^, ^101^ ^]^ and can even probe metal coordination in the solid state,^[^ ^102^ ^]^ although the low information content of typical ^31^P NMR experiments can, e.g., result in coincidentally identical spectra for different ^31^P nuclei.^[^ ^103^ ^]^ Although the calculation of NMR parameters is generally challenging, DFT‐calculated ^31^P chemical shifts for Ni‐phosphine complexes have been reported with reasonable accuracy.^[^ ^104^ ^]^ A combination of NMR spectroscopy, P VtC XES, and DFT would be a compelling program for future work.

### 2.6 Prospects for Catalysis

**Figure 9:**
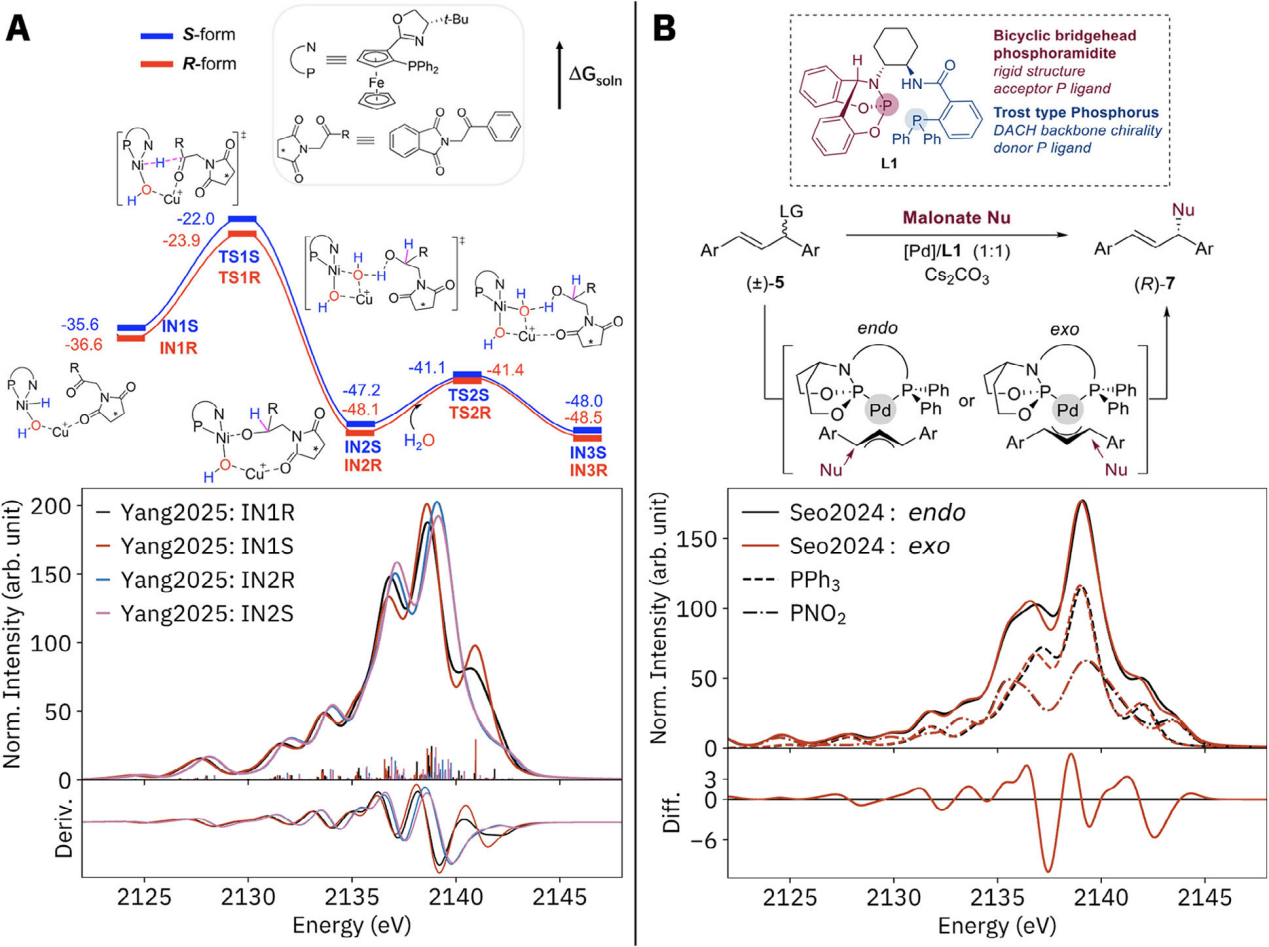
A) Calculated reaction pathway by Yang et al. for the asymmetric hydrogenation of amino ketones by a Ni‐Cu catalyst, with calculated P VtC XES spectra for the *R* and *S* intermediates before and after hydride attack at the ketone; B) catalytic scheme by Seo et al. for the asymmetric nucleophilic addition to diarylallyl substrates by a Pd catalyst, with calculated P VtC XES spectra for the two key *endo* and *exo* intermediates, including separate subspectra for the phosphine and phosphoramidite P sites (dashed and dot‐dashed lines). Reaction pathway in panel A adapted with permission from Yang et al. 2025; copyright 2025 American Chemical society. Scheme in panel B adapted with permission from Seo et al. 2024; copyright 2024 American Chemical Society.

To briefly demonstrate the potential for P VtC XES to differentiate highly similar species with different phosphorus‐containing ligands, calculated spectra are presented in **Figure** **9** for two recent asymmetric catalysis systems for which DFT reaction pathways and structures were reported.^[^ ^105^, ^106^ ^]^ To predict differentiability, spectral differences were quantified by the integrated absolute difference (IAD) between spectra (Table S3, Supporting Information). Experimental (calculated) IADs are typically smaller than those for calculated spectra, ranging from 119 (168) for Ni(dppp)Cl_2_ versus Ni(PPh_3_)_2_Cl_2_ to 48 (87) for Ni(dppp)Cl_2_ versus Ni(dppe)Cl_2_, although Ni(PPh_3_)_2_Br_2_ versus Ni(PPh_3_)_2_Cl_2_ was an exception at 58 (19).

The Ni─Cu asymmetric hydrogenation catalyst in Figure 9A features a large metalloligand with a phosphine coordinated to Ni.^[^ ^105^ ^]^ The enantiomers of two calculated intermediates, IN1, with a hydride/hydroxo Ni° center, and IN2, with an alkoxy/hydroxo Ni^0^, result in different spectra, with the greater difference found for the IN1 enantiomers (IAD = 72 and 32).^[^ ^105^ ^]^ From IN1 to IN2, the main P VtC peaks shift by +0.5 eV, a result of lower electron density at P and thus more stable P 1*s* orbitals in the latter complexes that have an alkoxy instead of hydride ligand at Ni. For the IN1R and IN1S phosphine sites, the most proximal conformational differences are a +0.02‐Å longer Ni─P bond in IN1S and the orientation of the hydroxide proton relative to the Ni coordination plane. Both the main peak and high‐energy shoulder are more intense for IN1S and result from MOs with significant P–Ni and Ni–H character, and higher P *p* character in the case of IN1S.

In Figure 9B, calculated spectra are presented for a palladium catalyst with a chiral bidentate ligand featuring phosphoramidite (formally P^3+^) and “Trost”‐type triphenylphosphine (formally P^3−^) moieties.^[^ ^106^ ^]^ The high enantioselectivity of this catalyst was attributed to the formation of *endo* and *exo* Pd‐diarylallyl adducts, the latter of which is favored by 3.8 kcal mol^−1^ and results in a planar coordination geometry. The two binding modes result in differentiable P VtC spectra (IAD = 39). Each phosphorus site contributes comparably to each total spectrum (53% phosphine, 47% phosphoramidite), with the phosphine subspectra similar to those measured here. The phosphoramidite P 1*s* orbitals are ‐1.7–1.9 eV lower in energy than those of the phosphines, reflecting the higher P oxidation state. Transitions from the HOMO of each complex (which have P lone‐pair, Pd 4*d*, and allyl π^nb^ character) to the two P 1*s* orbitals thus contribute to both high‐energy shoulders of each total spectrum. Although its total free energy is lower, the *exo* complex has a phosphine 1*s* orbital 0.2 eV higher in energy compared to that of the *endo* complex, resulting in a shift en masse of the *exo* spectrum to lower energy. This shift in P 1*s* indicates higher electron density at P, possibly resulting from a more effective donation from the diphenylallyl in the *exo* complex. The phosphine site is more sensitive to the diphenylallyl binding mode. Experimental investigation of P VtC XES for other phosphorus‐based ligands is ongoing.

## 3 Conclusion

Phosphine P VtC XES is a promising new spectroscopic tool for the characterization of catalysts, demonstrated here with a series of 5 closely related bisphosphine complexes and by comparison with Ni VtC XES. Difference spectra are well‐reproduced by DFT, which could facilitate the identification of unknown species by comparison with candidate spectra. Calculations of hypothetical models elucidate the physical origins of spectral variation, revealing sensitivity to metal oxidation and spin state, changes in complex conformation, and substitution of non‐phosphine ligands.

Compared to established X‐ray spectroscopic techniques, P VtC XES offers rich interpretable information content, accessible with a small natural linewidth and intense dipole‐allowed transitions. The technique is metal‐ and spin‐agnostic and readily applied to solids and frozen or flowing solutions. We expect that P VtC XES will prove most valuable in dissecting reaction mixtures, assigning conformations, and understanding more unusual electronic structures. The close connection between spectra and a ground‐state MO framework offers a compelling probe of electronic structure, which has promise for broad applications in homogeneous catalysis.

## Conflict of Interest

The authors declare no conflict of interest.

## Supporting information



Supporting Information

## Data Availability

All experimental and computational data, as well as example ORCA input files, are available at the Edmond open data repository: https://doi.org/10.17617/3.NINN3Q.
