# Supplementary material for: Combined X‐Ray Emission Spectroscopy at Phosphorus and Nickel: Detecting Subtle Changes in Catalyst Electronic Structure at High Resolution
Source: Small. 2025 Jun 26;21(37):2505199. doi: 10.1002/smll.202505199 (PMC12444823; doi:10.1002/smll.202505199)
Supplement: Supplementary file 1 — Supporting Information [file SMLL-21-2505199-s001.docx]

Combined X-ray Emission Spectroscopy at Phosphorus and Nickel: Detecting Subtle Changes in Catalyst Electronic Structure at High Resolution

Zachary Mathe, Serena DeBeer*

Table of Contents

[Complexes Studied 2](#_Toc195783319)

[Structure of Ni(PPh_3_)_2_Cl_2_ 2](#_Toc195783320)

[Experimental XES 3](#_Toc195783321)

[Data Collection 3](#_Toc195783322)

[Ni Kβ Mainlines 4](#_Toc195783323)

[Data Processing 5](#_Toc195783324)

[DFT Calculations 6](#_Toc195783325)

[General DFT Methods 6](#_Toc195783326)

[Ni VtC Calculation Methods 8](#_Toc195783327)

[Quantifying Differences 11](#_Toc195783328)

[Localization of MOs 12](#_Toc195783329)

[Ni(PPhMe_2_)_2_Cl_2_ Calculations 14](#_Toc195783330)

[Calculation Methods 15](#_Toc195783331)

[Ni(dppe)Cl_2_ and Ni(dppp)Cl_2_ 22](#_Toc195783332)

[Chloride Substitution 24](#_Toc195783333)

[References 26](#_Toc195783334)

# Complexes Studied

All chemicals were purchased in the highest available purity from Merk/Sigma and used without dilution or further purification.

## Structure of Ni(PPh_3_)_2_Cl_2_

The complex Ni(PPh_3_)_2_Cl_2_ has been characterized in two distinct isomers: tetrahedral (*S* = 1) and square-planar with *trans* triphenylphosphines (*S* = 0).^[1–5]^ Which isomers are present in a solution or crystal depends on the choice of solvent(s) used. The two isomers are readily distinguished by color: the tetrahedral complex has been described as dark blue or green, while the square-planar complex is red. In solvent-free crystals, Ni(PPh_3_)_2_Cl_2_ adopt the tetrahedral conformation, and drying of *trans* square-planar crystals *in vacuo* restores the tetrahedral conformation when solvent is removed.^[5]^ To confirm that the Ni(PPh_3_)_2_Cl_2_ used in the present study was tetrahedral, the powder was inspected under a microscope and found to be highly homogeneous, with well-defined crystals with an intense blue color.

# Experimental XES

## Data Collection

P and Ni Kβ X-ray emission data were collected and processed following previously published procedures at the PINK beamline at the BESSY II synchrotron.^[6–8]^ Sample cells were constructed from 1-mm aluminum spacers and 4-μm high-purity polypropylene film windows (SPEX SamplePrep, “Ultralene”) using double-sided tape (Tesa, “universal white”). The backs of samples were sealed with polyimide tape (DuPont, “Kapton”). All samples were prepared and sealed in a nitrogen glovebox and transferred into the sample measurement chamber in ambient atmosphere. Samples were measured at ambient temperature and a helium atmosphere of 10 mbar. Spectra were collected for about 20 minutes for P and 40 minutes for Ni.

Phosphorus VtC X-ray emission was measured with a vacuum von Hamos spectrometer equipped with a Si(111) cylindrical bent crystal with a radius of 250 mm, the same apparatus used for a previous P VtC XES study.^[6]^ The incident beam had an energy of 4,000 eV with a FWHM bandpass of ~80 eV. The energy was calibrated to the P VtC spectrum of NaH_2_PO_4_ with peak energies of 2139.5, 2137.9, 2135.3, and 2123.4 eV.^[6,9]^

Nickel Kβ X-ray emission, including the mainline and VtC, was measured with an ambient-pressure von Hamos spectrometer equipped with a diced Si(111) crystal (using the Si(444) reflection) with a radius of 250 mm, the same apparatus used for a previous Ni Kβ XES study.^[7]^ The incident beam had an energy of 9500 keV with a FWHM bandpass of ~100 eV. The energy was calibrated using a set of metal foil emission lines: Ta Lα_1_ (8146.1 eV), Ni Kβ (8264.66 eV), and Tb Lα_1,2_ (6272.8 and 6238.0 eV; Si(333) reflection).^[7]^ We note that the relatively low point-density of the present Ni VtC spectra could be increased with a focusing spectrometer geometry and the signal-to-noise would improve with longer measurements.

The present samples were all prepared in 1-mm thickness because they are readily available. Sample material usage could be greatly reduced for P VtC, with no loss of signal, by using much thinner samples, whether powder or solution, because the attenuation length of P VtC emission is very short (e.g. 20 μm in frozen water). Thinner cells have been developed to allow the collection of P VtC spectra from as little as 15 μL of a 40 mM (in P atoms) solution with no reduction of signal-to-noise, facilitating the measurement of catalytically relevant materials

## Ni Kβ Mainlines

The full Ni Kβ spectra, including the mainlines and VtC, are presented in Figure S1.


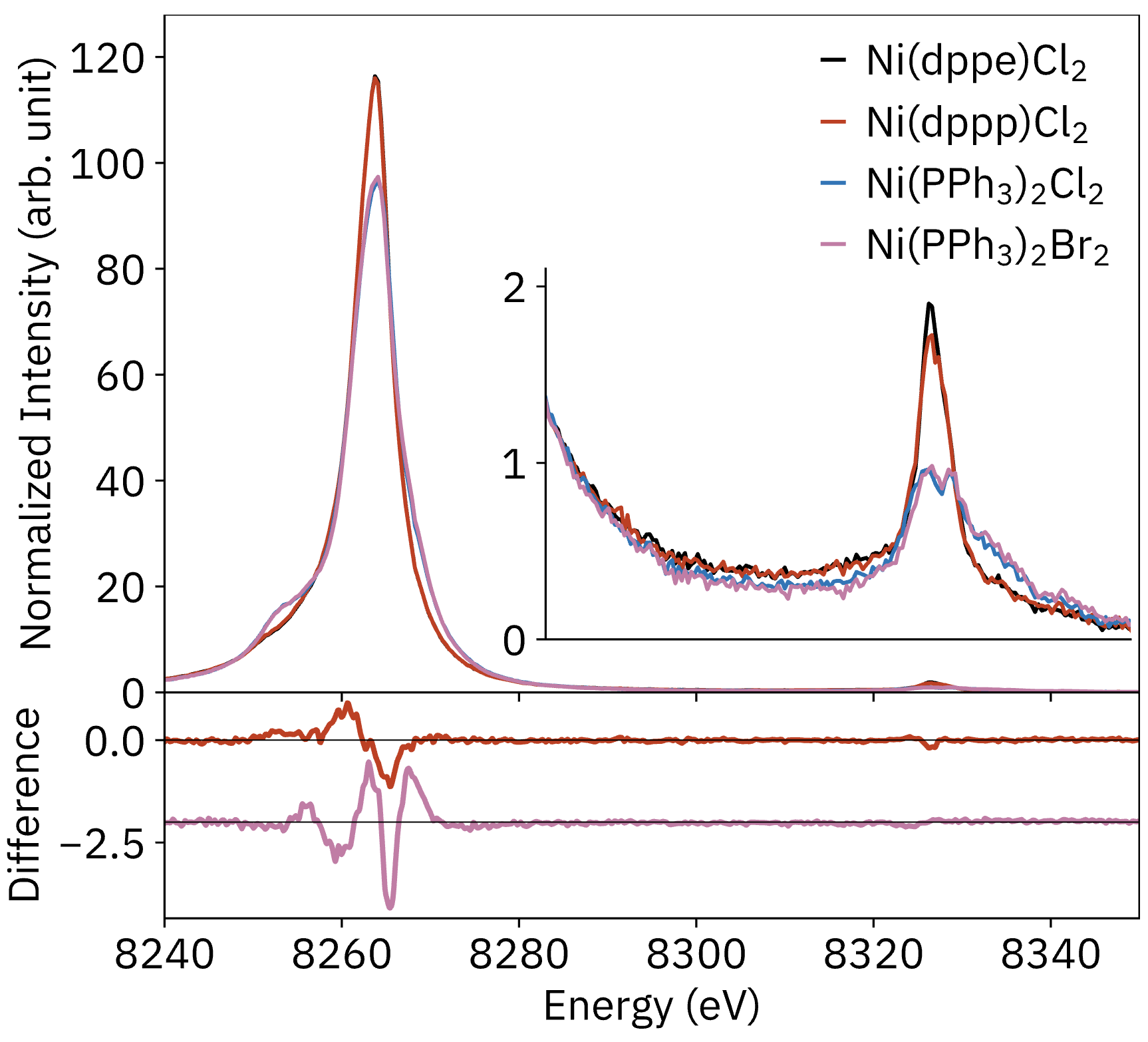


Figure S1. Full Ni Kβ XES of the four nickel complexes, normalized to a total area of 1000. The VtC region is re-plotted inset on the same energy axis but with a 35x y-axis. Difference spectra are plotted below for Ni(dppp)Cl_2_ – Ni(dppe)Cl_2_ (red) and for Ni(PPh_3_)_2_Br_2_ – Ni(PPh_3_)_2_Cl_2_ (pink).

## Data Processing

All XES spectra were baseline-corrected (Figure S2). For P XES, a linear baseline was used, while for Ni a cubic baseline was used. Baselines were fitted to featureless regions of equal size on either side of the Kβ emission features.


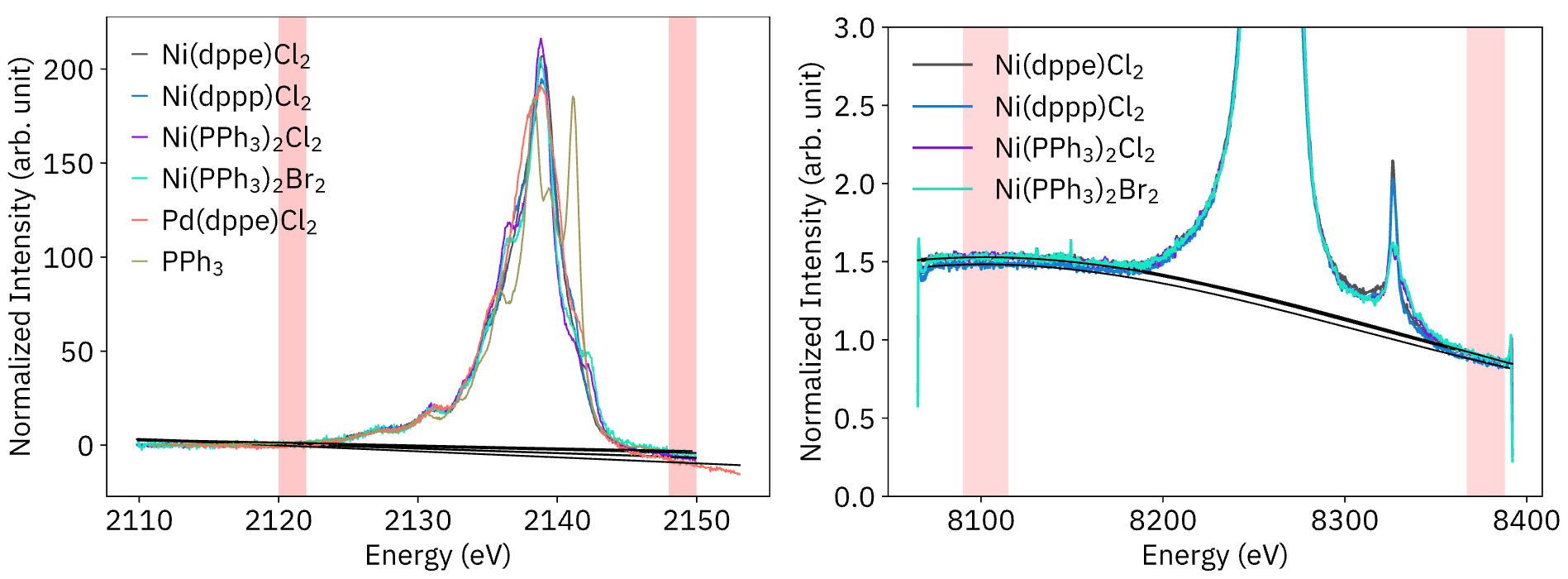


Figure S2. Baseline fitting of PXES (left) and Ni XES (right), with the fitted regions marked in pink and the fitted baselines in black.

Because the spectrometer greatly oversamples the spectra (CCD resolution of ~0.036 eV/pixel,^[6]^ versus P 1*s* lifetime of 0.47 eV^[10]^), spectra were conservatively smoothed with a Whittaker-Eilers smoother,^[11]^ as implemented in the python package whittaker-eilers (Figure S3).^[12]^ The smoothing parameter λ was chosen for each spectrum by minimization of the root cross validation error. The Ni XES was not smoothed.


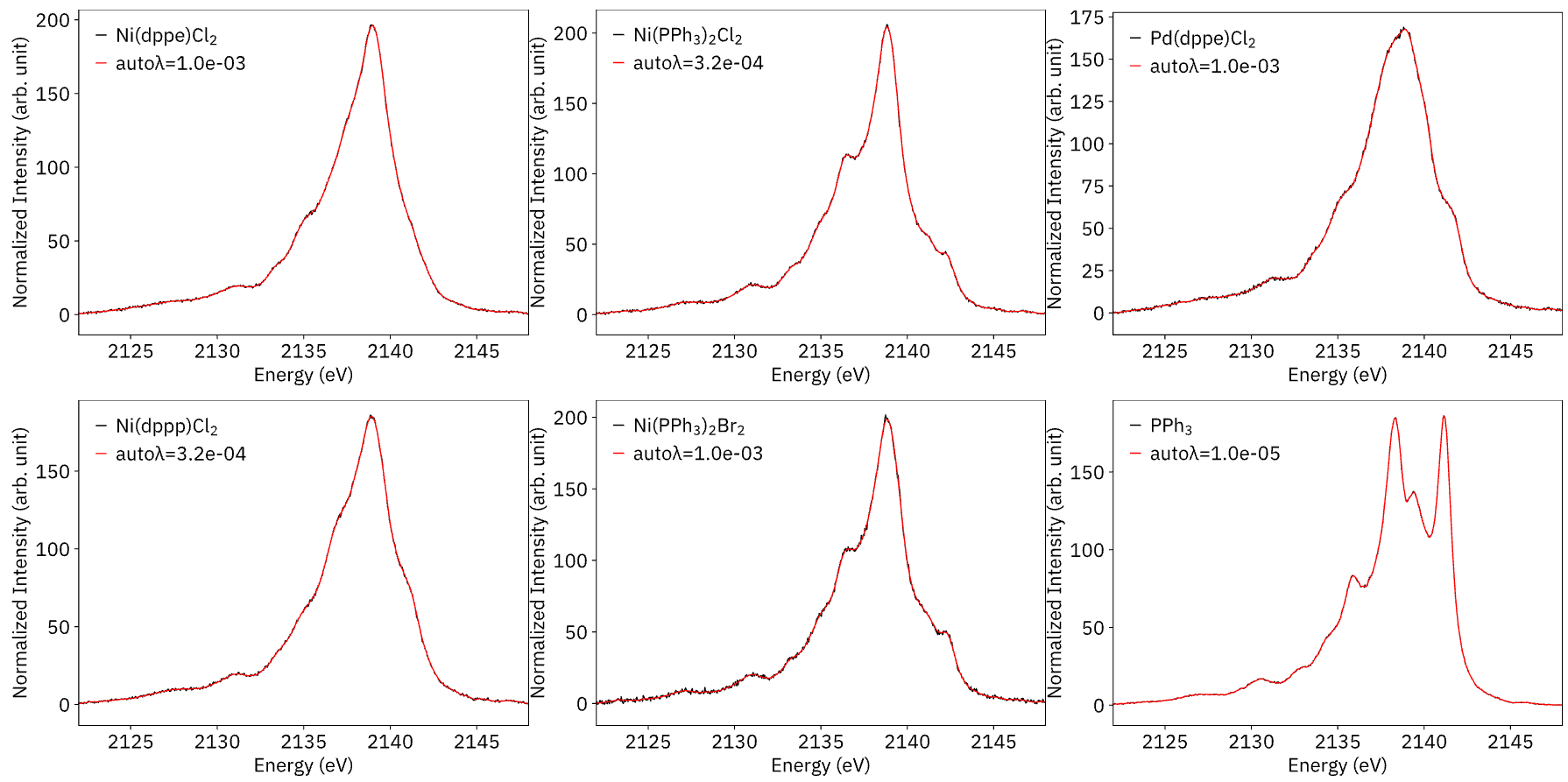


Figure S3. Unsmoothed (black) and smoothed (red) P Kβ XES, including the optimized smoothing parameter λ for each spectrum.

# DFT Calculations

## General DFT Methods

All density functional theory (DFT) calculations were performed with ORCA v6.0.1, except the VtC spectra calculated with TD-DFT and the FFMIO operator, which were performed with a local development version of ORCA (6.0.x, 2025-03-31) as a result of a limitation in the release version.^[13]^ Unrestricted determinants, the TPSSh metahybrid functional,^[14]^ the X2C approximation for relativistic effects, the D3BJ dispersion correction,^[15,16]^ and a conductor-like polarizable continuum model (CPCM, with an infinite dielectric) of solvation^[17,18]^ were used. x2c-TZVPall basis sets were used for all elements and the RIJCOSX approximation was used with x2c/J auxiliary basis sets.^[19]^ All spectra were calculated for optimized molecular geometries. Wavefunctions were analyzed with Multiwfn.^[20,21]^

Additional calculations were performed to determine the effects of the density functional, solvation, reference orbitals and transition calculation method on the performance of P VtC XES calculations. Spectra calculated with the PBE0 hybrid functional^[22]^ were of similar quality to those from TPSSh, while the difference spectrum calculated with the r2SCAN meta-GGA functional^[23]^ for Ni(PPh_3_)_2_Br_2_ versus Ni(PPh_3_)_2_Cl_2_ was notably less accurate. The inclusion of CPCM solvation was also found to be essential for the Ni(PPh_3_)_2_Br_2_ versus Ni(PPh_3_)_2_Cl_2_ difference spectrum, and is also expected to be important for any systems with negative or varying total charges. All structures and orbitals are plotted with orange for P, light green for Cl, burgundy for Br, green for Ni, teal for Pd, grey for C, and with all H omitted for clarity. The use of quasi-restricted orbitals^[24]^ (QROs) instead of canonical orbitals for the open-shell species resulted in less accurate difference spectra.

Various approaches were investigated for the calculation of XES spectra. Two methods were used for the actual transition calculations: the one-electron-one-orbital (1e1o) method, in which transition energies and intensities are obtained from the relative energies and dipole overlaps of canonical orbitals, and time-dependent DFT (TD-DFT) with core-valence separation. The 1e1o method is computationally inexpensive, requires little user input, and is well-established for transition metal valence-to-core XES, including Ni.^[7,25–27]^ TD-DFT may somewhat account for multi-excitation character and effects of the hole in the final state, which has been reported to improve valence-to-core spectra.^[28,29]^ For each transition method, two different reference wavefunctions were used: the typical ground-state (GS) wavefunction and a core-hole wavefunction, obtained by ΔSCF,^[30,31]^ both of which have been used to calculate valence-to-core spectra.^[25,29,32]^ Additionally, for the open-shell complexes, both canonical MOs and quasi-restricted orbitals (QROs) were used to calculate transitions. P VtC XES in particular has been reported using the ΔSCF/TD-DFT^[33]^ as well as GS/1e1o^[6]^ methods.

Spectra were calculated from transitions using a Voigt lineshape with Gaussian and Lorentzian broadenings σ = 0.46 eV and γ = 0.24 eV (HWHM). The value of γ corresponds to the P 1*s* lifetime of 0.47 eV FWHM.^[10]^ The value of σ was chosen to most clearly show the different spectral features; a closer visual approximation of the experimental spectra could be obtained with a higher Gaussian broadening (see SI). Except where otherwise noted, all calculated P VtC spectra are shifted by a constant value of +47.9 eV to align best with experiment. Sticks for each transition are plotted with a different scaling for each species for visual clarity.

## Ni VtC Calculation Methods

All Ni VtC spectra and calculations are shown in Figure S4. All spectra were normalized, including the entire Kβ region (mainline + VtC), to an area of 1000. The 1e1o and TD-DFT methods provide quite different spectra, with the 1e1o perhaps more accurately capturing the two main peaks of the tetrahedral Ni(PPh_3_)_2_X_2_ complexes. The interpretation of the results is complicated by transitions within and above the Ni pre-edge (approximately 8333.3 eV^[34]^) that result from intermediate states with multiple core holes.^[7,35]^ A more thorough interpretation of these spectra and their calculation is beyond the scope if this work.

Three light-matter interaction operators were used to calculate transition intensities between orbitals (in the 1e1o method) and excited states (for TD-DFT):

- the standard dipole operator, which produces .XES.stk and .ABS.stk files in ORCA
- the older “origin-adjusted” (OA) method, introduced in ORCA more than a decade ago,^[36]^ which produces .XESQ.stk and .ABSQ.stk files in ORCA
- the newer full (semiclassical) field-matter interaction operator (FFMIO),^[37,38]^ introduced in ORCA 6, which produces FFMIO.stk files in ORCA

For P VtC XES, all operators produced identical spectra, confirming that the transitions are fully dipole-allowed without any e.g. electric quadrupole contributions, as expected from their essential P 3*p* → 1*s* character.

For Ni VtC XES, all three operators produced different spectra (Figure S4), although the variation is smaller than the variation between 1e1o versus TD-DFT methods. For the 1e1o methods, the dipole and FFMIO spectra were most similar, while for the TD-DFT methods, the dipole and origin-adjusted spectra were most similar. Intensity ratios are provided in Table S1 and differences between intensity-weighted average energies (IWAEs) are provided in Table S2

These calculations show that the dipole approximation of the full light-matter interaction can introduce error for Ni VtC transitions and thus the more accurate FFMIO method should be preferred. This shortcoming can be understood as resulting from Ni 3*d→*1*s* character in the transitions, which is dipole-forbidden but quadrupole-allowed. The FFMIO method uses a much more accurate approximation of the actual light-matter interaction compared to the origin-adjusted method; thus, the present results indicate significant shortcomings of the origin-adjustment method for calculating Ni VtC transitions. We also note that the experimental relative heights of the main Ni VtC peaks for Ni(dppe)Cl_2_ versus Ni(dppp)Cl_2_ are better-reproduced than the FFMIO method.

VtC XES has been reported to have significant quadrupole contributions for Cu^[39]^ and Zn.^[40]^ The present results strongly support the use of a FFMIO for the calculation of late 3*d* metal VtC.


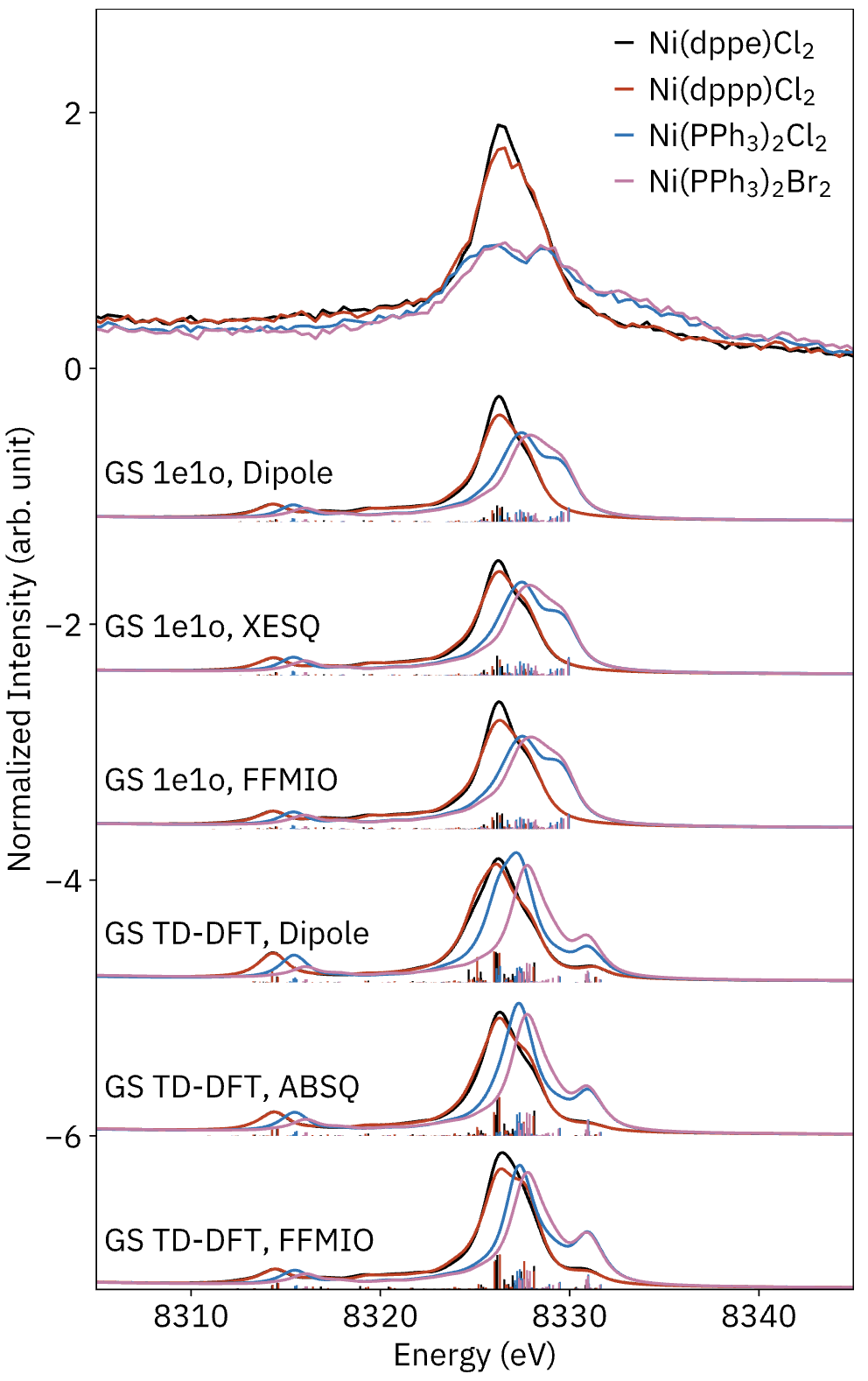


Figure S4. Ni VtC spectra (top), with calculations below, with the beyond-dipole calculations labeled according to the output of the orca_mapspc module of ORCA.

Table S1. Integrated intensities of the Ni Kβ_2,5_ region (integrated over [8310, 8340] eV) for six calculation methods (1e1o and TD-DFT transitions; dipole, origin-adjusted (OA) and full field-matter interaction (FFMIO) operators). Also included are intensity ratios of different techniques for each species (below), as well as between species for each technique (right).

| (arb. unit) | Ni(dppe)Cl_2_ | Ni(dppp)Cl_2_ | Ni(PPh_3_)_2_Cl_2_ | Ni(PPh_3_)_2_Br_2_ | dppp /dppe | (PPh_3_)_2_ /dppp | Br_2_ /Cl_2_ |
| --- | --- | --- | --- | --- | --- | --- | --- |
| 1e1o Dipole | 15.458 | 15.126 | 14.080 | 13.483 | 0.979 | 0.931 | 0.958 |
| 1e1o OA | 14.607 | 14.693 | 14.504 | 13.844 | 1.006 | 0.987 | 0.955 |
| 1e1o FFMIO | 15.955 | 15.608 | 14.620 | 14.116 | 0.978 | 0.937 | 0.966 |
| TD-DFT Dipole | 17.206 | 17.353 | 17.430 | 15.648 | 1.009 | 1.004 | 0.898 |
| TD-DFT OA | 16.400 | 16.708 | 16.801 | 16.037 | 1.019 | 1.006 | 0.955 |
| TD-DFT FFMIO | 18.455 | 18.053 | 16.848 | 16.253 | 0.978 | 0.933 | 0.965 |
|  |  |  |  |  |  |  |  |
| 1e1o Dipole/FMIO | 0.969 | 0.969 | 0.963 | 0.955 |  |  |  |
| 1e1o OA/FFMIO | 0.916 | 0.941 | 0.992 | 0.981 |  |  |  |
| TD-DFT Dipole/FFMIO | 0.932 | 0.961 | 1.035 | 0.963 |  |  |  |
| TD-DFT OA/FFMIO | 0.889 | 0.925 | 0.997 | 0.987 |  |  |  |

Table S2. Intensity-weighted average energies (IWAEs), in eV, of the Kβ_2,5_ region (integrated over [8310, 8340] eV) for six calculation methods (1e1o and TD-DFT transitions; dipole, origin-adjusted (OA) and full field-matter interaction (FFMIO) operators). Also included are IWAE differences of different techniques for each species (below), as well as between species for each technique (right).

| (eV) | Ni(dppe)Cl_2_ | Ni(dppp)Cl_2_ | Ni(PPh_3_)_2_Cl_2_ | Ni(PPh_3_)_2_Br_2_ | dppp - dppe | (PPh_3_)_2_ - dppp | Br_2_ - Cl_2_ |
| --- | --- | --- | --- | --- | --- | --- | --- |
| 1e1o Dipole | 8324.592 | 8324.599 | 8325.968 | 8326.271 | 0.007 | 1.369 | 0.303 |
| 1e1o OA | 8324.595 | 8324.613 | 8325.985 | 8326.297 | 0.018 | 1.372 | 0.312 |
| 1e1o FFMIO | 8324.696 | 8324.701 | 8326.070 | 8326.365 | 0.005 | 1.369 | 0.295 |
| TD-DFT Dipole | 8324.580 | 8324.554 | 8325.638 | 8326.394 | -0.026 | 1.084 | 0.756 |
| TD-DFT OA | 8324.688 | 8324.688 | 8325.984 | 8326.428 | 0.000 | 1.296 | 0.444 |
| TD-DFT FFMIO | 8324.917 | 8324.919 | 8326.296 | 8326.582 | 0.002 | 1.377 | 0.286 |
|  |  |  |  |  |  |  |  |
| 1e1o Dipole - FMIO | -0.104 | -0.102 | -0.102 | -0.094 |  |  |  |
| 1e1o OA - FFMIO | -0.101 | -0.088 | -0.085 | -0.068 |  |  |  |
| TD-DFT Dipole - FFMIO | -0.337 | -0.365 | -0.658 | -0.188 |  |  |  |
| TD-DFT OA - FFMIO | -0.229 | -0.231 | -0.312 | -0.154 |  |  |  |

## Quantifying Differences

Differences between pairs of spectra (either two experimental or two calculated) were quantified by their pair integrated absolute difference (IAD), i.e. the integral of the absolute value of one spectrum subtracted from the other. IADs for various systems are presented in Table S3. A fairly consistent overestimation of difference is found for the DFT versus experimental spectra, with the notable exception of Ni(PPh_3_)_2_Br_2_ versus Ni(PPh_3_)_2_Cl_2_, for which the difference is underestimated by DFT. IADs do not measure the accuracy of difference spectra, only their differentiability.

Table S3. Pair integrated absolute differences for various experimental and calculated spectra.

|  | **Experiment** | **DFT (GS 1e1o)** | **DFT - Exp** |
| --- | --- | --- | --- |
| **This Work** |  |  |  |
| Ni(dppp)Cl_2_ - Ni(dppe)Cl_2_ | 48 | 87 | 39 |
| Ni(dppe)Cl_2_ - Ni(PPh_3_)_2_Cl_2_ | 115 | 169 | 54 |
| Ni(dppp)Cl_2_ - Ni(PPh_3_)_2_Cl_2_ | 119 | 168 | 48 |
| Ni(PPh_3_)_2_Br_2_ - Ni(PPh_3_)_2_Cl_2_ | 58 | 19 | -40 |
| Pd(dppe)Cl_2_ - Ni(dppe)Cl_2_ | 70 | 113 | 43 |
|  |  |  |  |
| **Yang2025**^[41]^ |  |  |  |
| IN1R - IN1S |  | 72 |  |
| IN2R - IN2S |  | 32 |  |
| IN2R - IN1R |  | 198 |  |
| IN2S - IN1S |  | 247 |  |
|  |  |  |  |
| **Seo2024**^[42]^ |  |  |  |
| *endo*-Pd-L – *exo*-Pd-L |  | 39 |  |
|  |  |  |  |
| **Harlow1983**^[43]^ |  |  |  |
| Opt Co(I) - Co(II) |  | 157 |  |

## Localization of MOs

In coordination complexes such as those studied here, electronic structure is typically understood in terms of chemical bonds: in each phosphine moiety, the phosphorus has one σ bond to each phenyl or alkyl carbon, and in the coordination complexes a fourth dative σ bond is formed to the metal by formal mixing of the phosphine lone pair with the metal valence *d* orbital(s). These familiar local bonding orbitals are readily identified in the DFT wavefunction by e.g. Pipek-Mezey localization (Figure S5).

The calculation of VtC spectra by a 1e1o method necessarily relies on the canonical MOs because transitions energies are calculated as the difference in orbital energies. Canonical MOs, however, are maximally delocalized and can be difficult to interpret, a problem that becomes worse the larger the system under investigation.^[44]^ The lack of correspondence between localized bonding orbitals and valence canonical MOs can be illustrated by their overlap (Figure S6).^[20]^ Deeper-lying core and semi-core orbitals (canonical MOs numbered <~55 in this case) are spanned by a relatively small number of atomic-like localized MOs. On the other hand, valence canonical MOs are generally spanned by a mixture of localized MOs and thus often cannot be efficiently and accurately described using the terminology of chemical bonding.


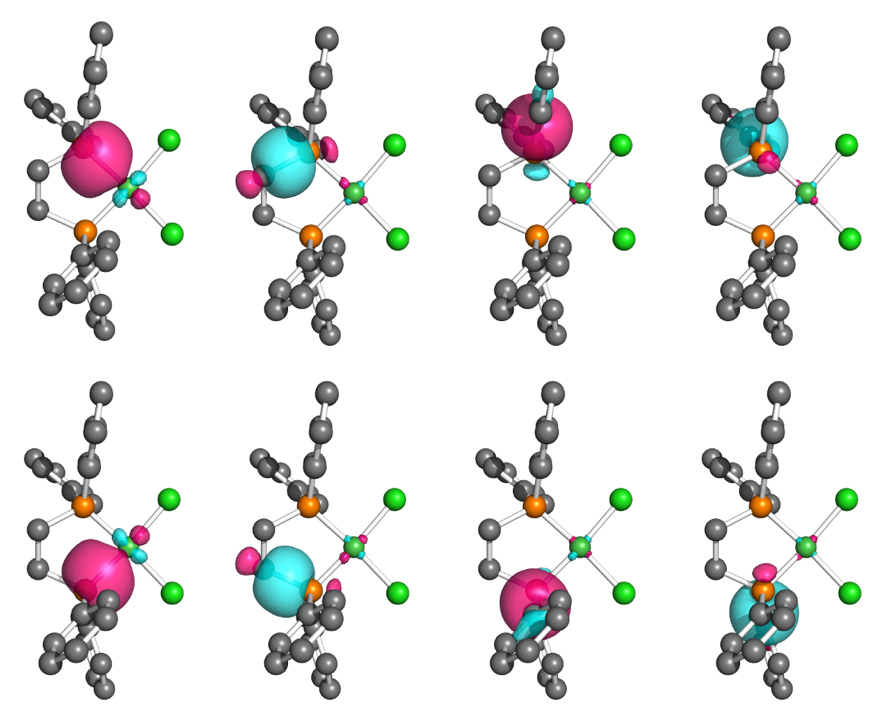


Figure S5. Pipek-Mezey localized orbitals for Ni(dppe)Cl_2_ identified as σ bonds with phosphorus.


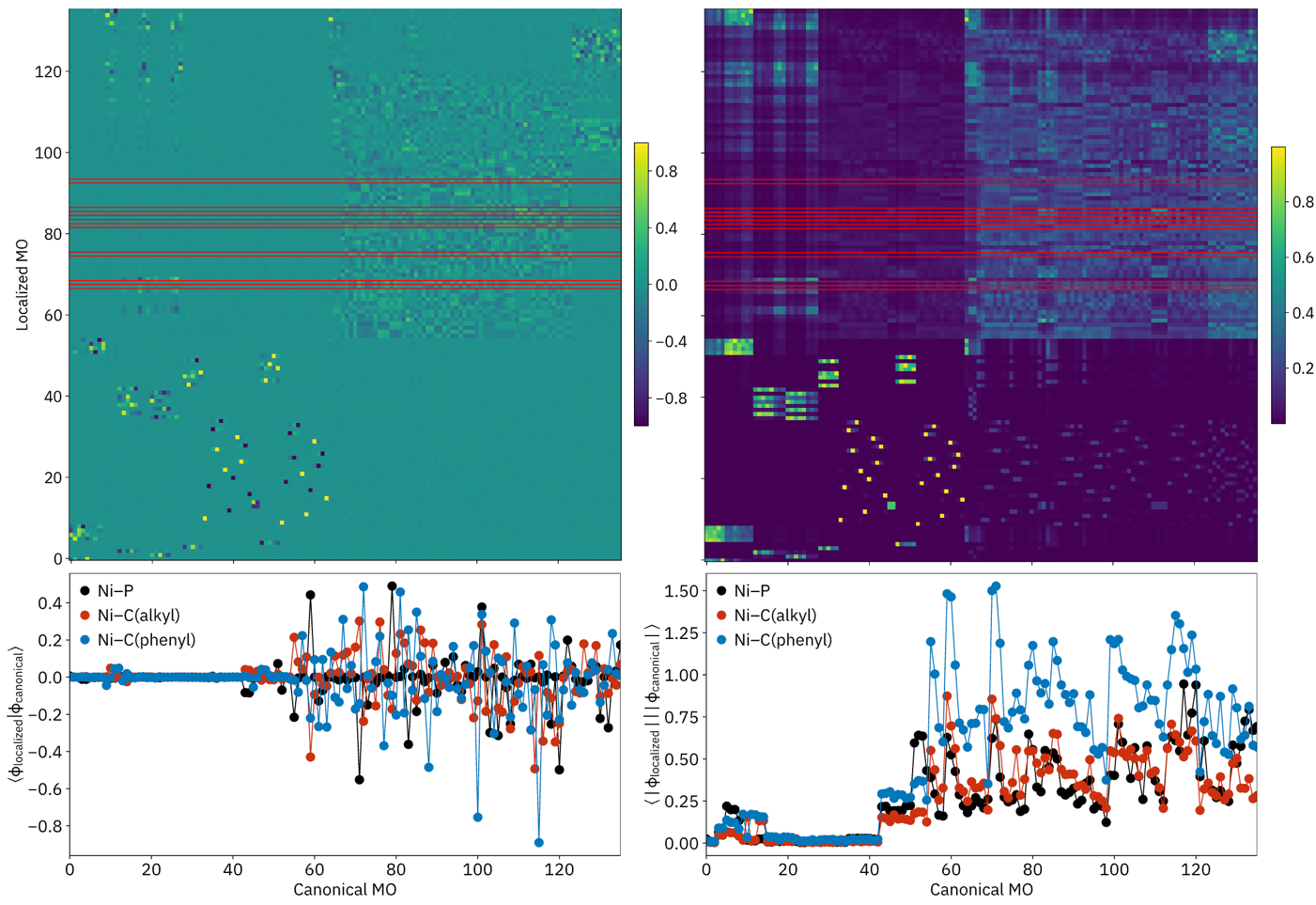


Figure S6. Overlaps between the occupied localized and canonical MOs, calculated either by $\left\langle\varphi_{local} | \varphi_{canonical} \right\rangle$ (left) or by $\left\langle\left| \varphi_{local} \right| | \left| \varphi_{canonical} \right| \right\rangle$ (right), with the eight localized orbitals identified as σ bonds with phosphorus bracketed. Below, the summed overlaps for each type of localized orbital with each canonical MO are plotted. The highest-numbered (124-135) canonical/localized MOs are more block-diagonal that the rest of the valence; these correspond to phenyl π localized MOs, which have significant overlap with the highest-energy canonical MOs.

## Ni(PPhMe_2_)_2_Cl_2_ Calculations

The sensitivity of P Kβ XES to ligand conformation was explored using Ni(PPhMe_2_)_2_Cl_2_. Thanks to the small methyl ligands, this complex is conformationally flexible without much opportunity for confounding ligand-ligand interactions. The three conformations shown in color in the main text are plotted larger and colored by element in Figure S7, and their energies are provided in Table S4. Also plotted in Figure S7 are the donor MOs for the most intense transitions of the highest-energy P Kβ emission features, which have dominantly P(lone-pair) and Cl(3*p*) character. For conformations B and C, the phenyl C(2*p*) orbitals are oriented towards the phosphorus lone-pairs, and these MOs also have phenyl(π*) character.


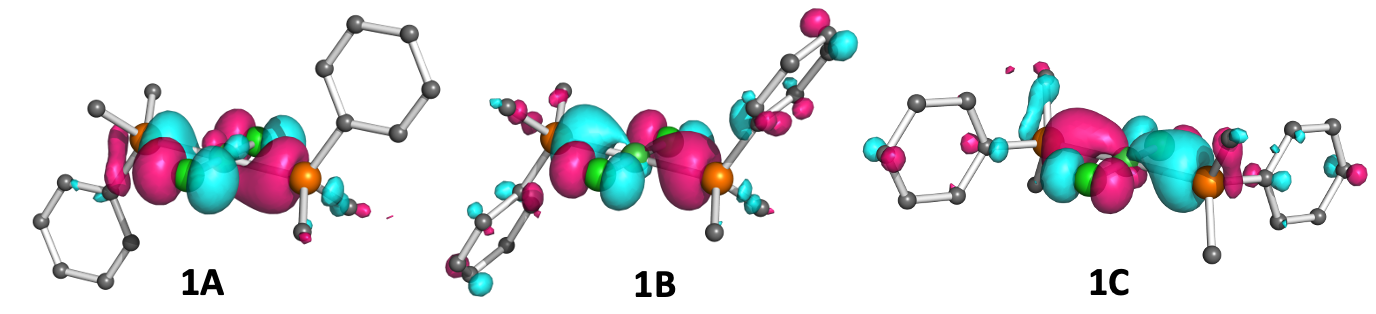


Figure S7. Three conformations of Ni(PPhMe_2_)_2_Cl_2_, A–C, including the donor MOs for the most intense transitions of the highest-energy P Kβ emission features at 2141.0–2141.3 eV.

Table S4. Calculated energies of conformers Ni(PPhMe_2_)_2_Cl_2_, including the SCF electronic energy and the Final Single Point Energy (which includes the D3 dispersion correction), as well as energy differences.

|  | SCF Energy (kcal/mol) | Final Single Point Energy (kcal/mol) |
| --- | --- | --- |
| A | -2354417.78 | -2354470.92 |
| B | -2354418.07 | -2354471.00 |
| C | -2354422.07 | -2354475.52 |
| B - A | -0.29 | -0.08 |
| B - C | 4.01 | 4.52 |

It was hypothesized that the ligand conformation could affect Ni–P π back-bonding; however, no clear evidence of this was found in the calculations. One signature of back-bonding would shorter Ni–P bonds and longer P–C bonds, but in these calculations, only small, positively correlated changes in distances were found. A natural orbitals for chemical valence (NOCV) analysis was also performed, but among the orbital pairs with a pair energy greater than 1 kcal/mol, no Ni–P π interactions were found.

## Calculation Methods

Spectra were calculated using the r2SCAN and B3LYP functionals, in addition to the TPSSh calculations shown in the main text (Figure S8 and Figure S9). The meta-GGA r2SCAN performed worse for the difference spectrum of the Ni(PPh_3_)_2_X_2_.


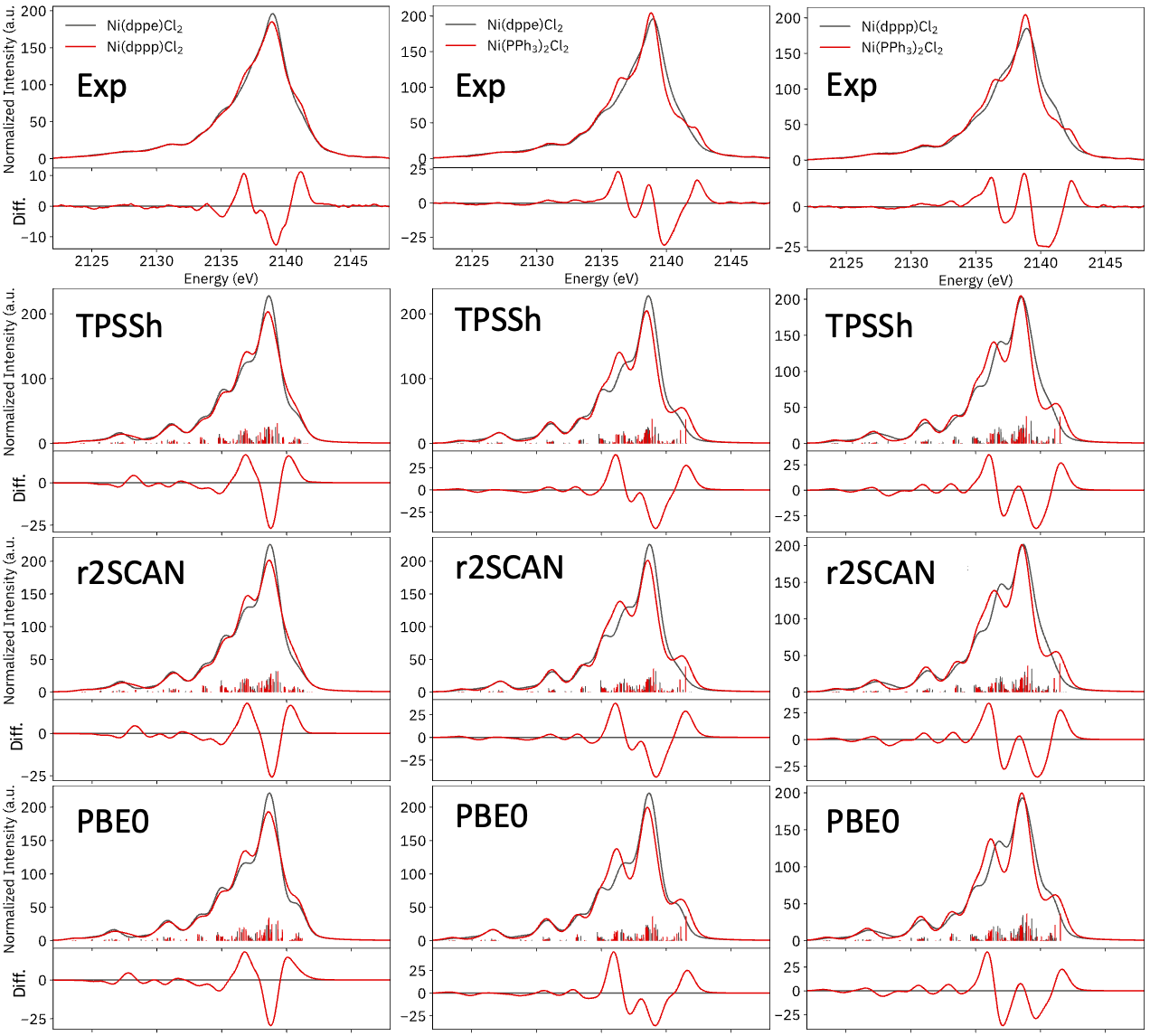


Figure S8. Calculations using the GS 1e1o method and the TPSSh, B3LYP, and r2SCAN functionals, presented with difference spectra (red – black), for Ni(dppe)Cl_2_, Ni(dppp)Cl_2_, and Ni(PPh_3_)_2_Cl_2_.


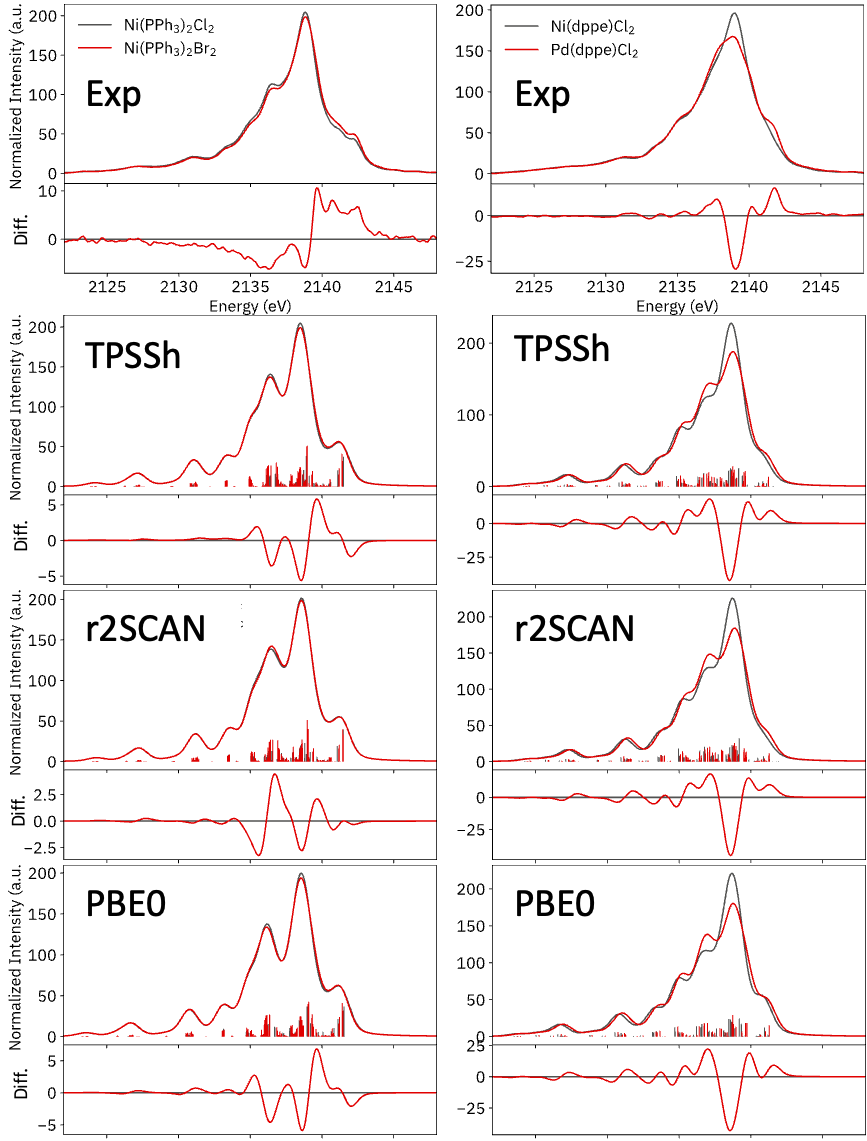


Figure S9. Calculations using the GS 1e1o method and the TPSSh, B3LYP, and r2SCAN functionals, presented with difference spectra (red – black), for Ni(dppe)Cl_2_, Pd(dppe)Cl_2_, Ni(PPh_3_)_2_Cl_2_. and Ni(PPh_3_)_2_Br_2_.

The inclusion of CPCM solvation significantly improved the difference spectrum of the Ni(PPh_3_)_2_X_2_, and is expected to be important for any calculations involving species of negative or differing total charge (Figure S10 and Figure 11).


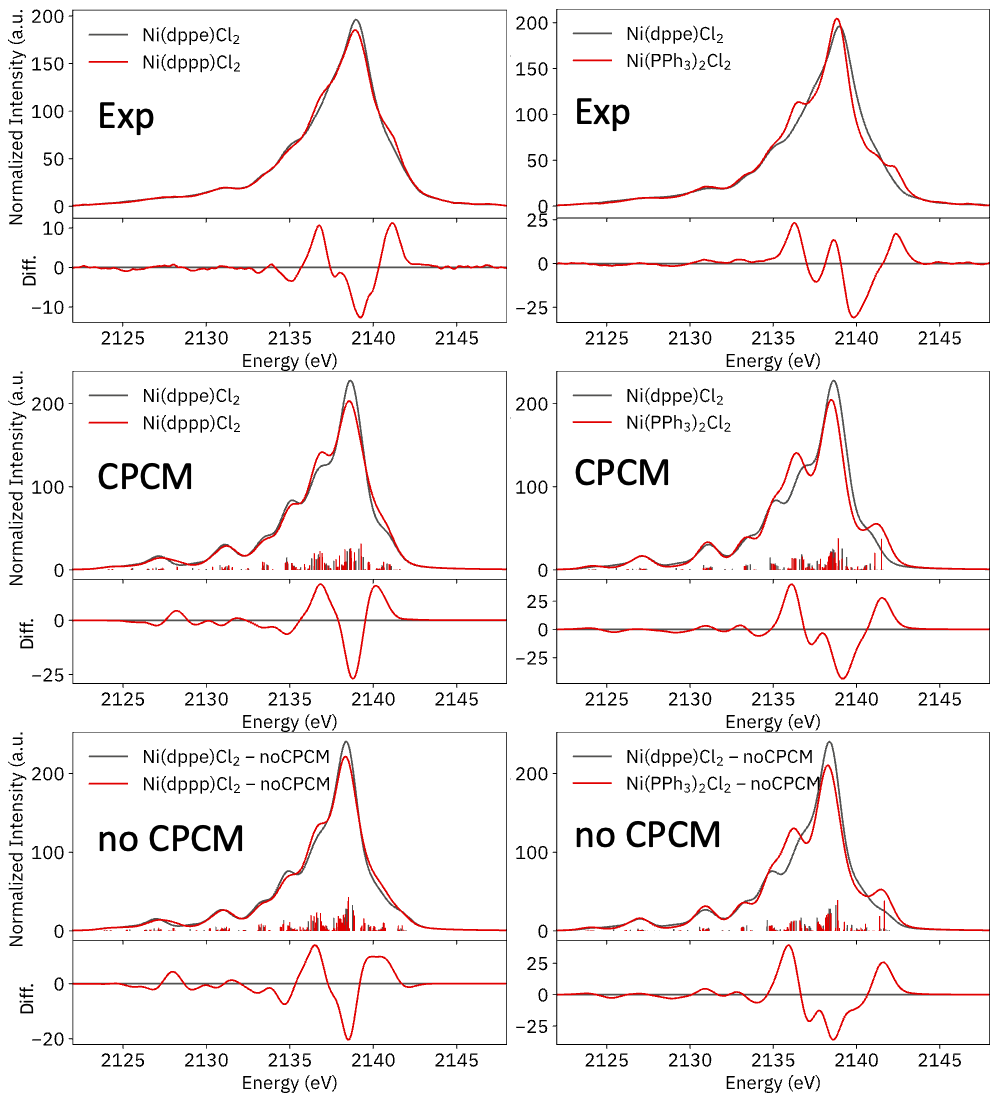


Figure S10. Calculations using the GS 1e1o method, with and without CPCM solvation, presented with difference spectra (red – black), for Ni(dppe)Cl_2_, Ni(dppp)Cl_2_, and Ni(PPh_3_)_2_Cl_2_.


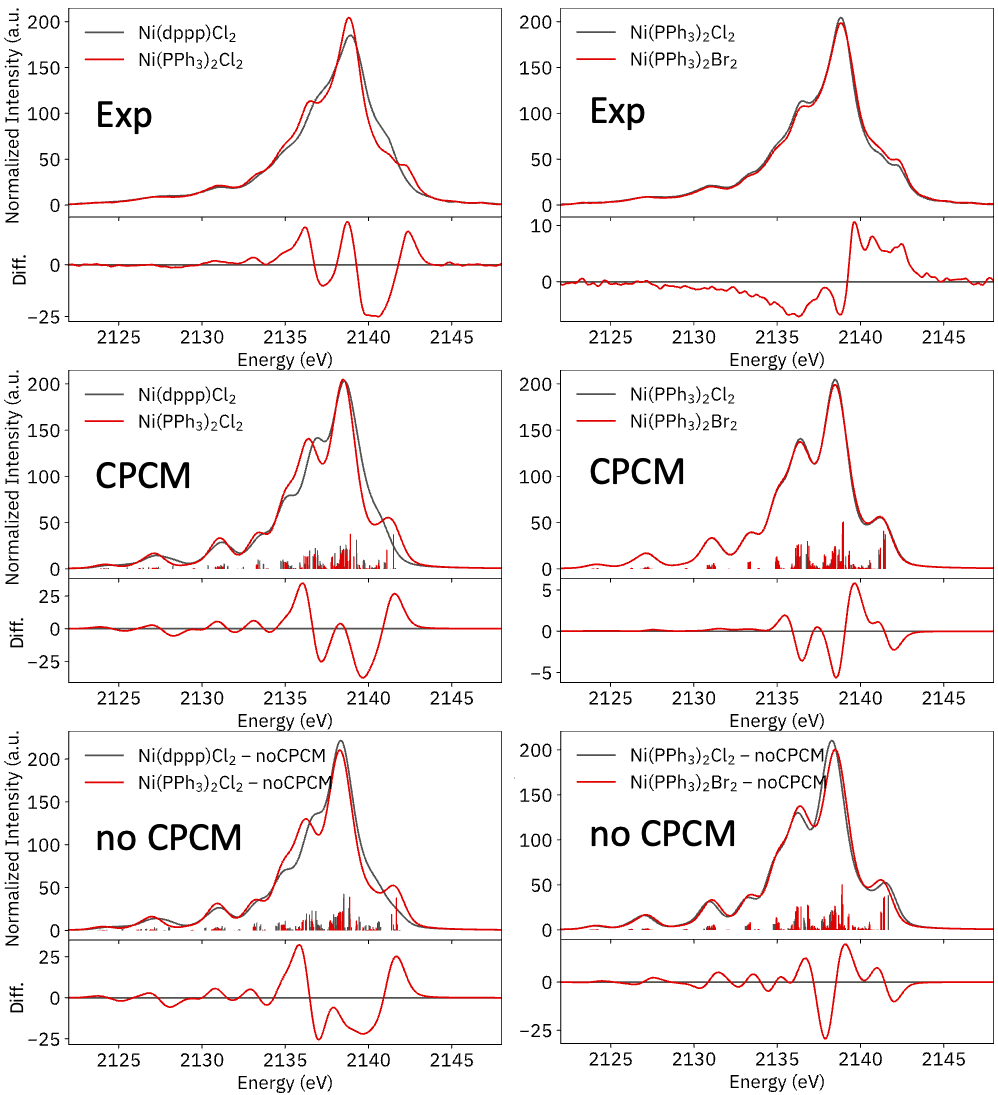


Figure 11. Calculations using the GS 1e1o method, with and without CPCM solvation, presented with difference spectra (red – black), for Ni(dppp)Cl_2_, Ni(PPh_3_)_2_Cl_2_, and Ni(PPh_3_)_2_Br_2_.

The use of quasi-restricted orbitals^[24]^ (QROs) in the GS 1e1o method for the open-shell species, instead of canonical orbitals, resulted in less accurate difference spectra (Figure S12).


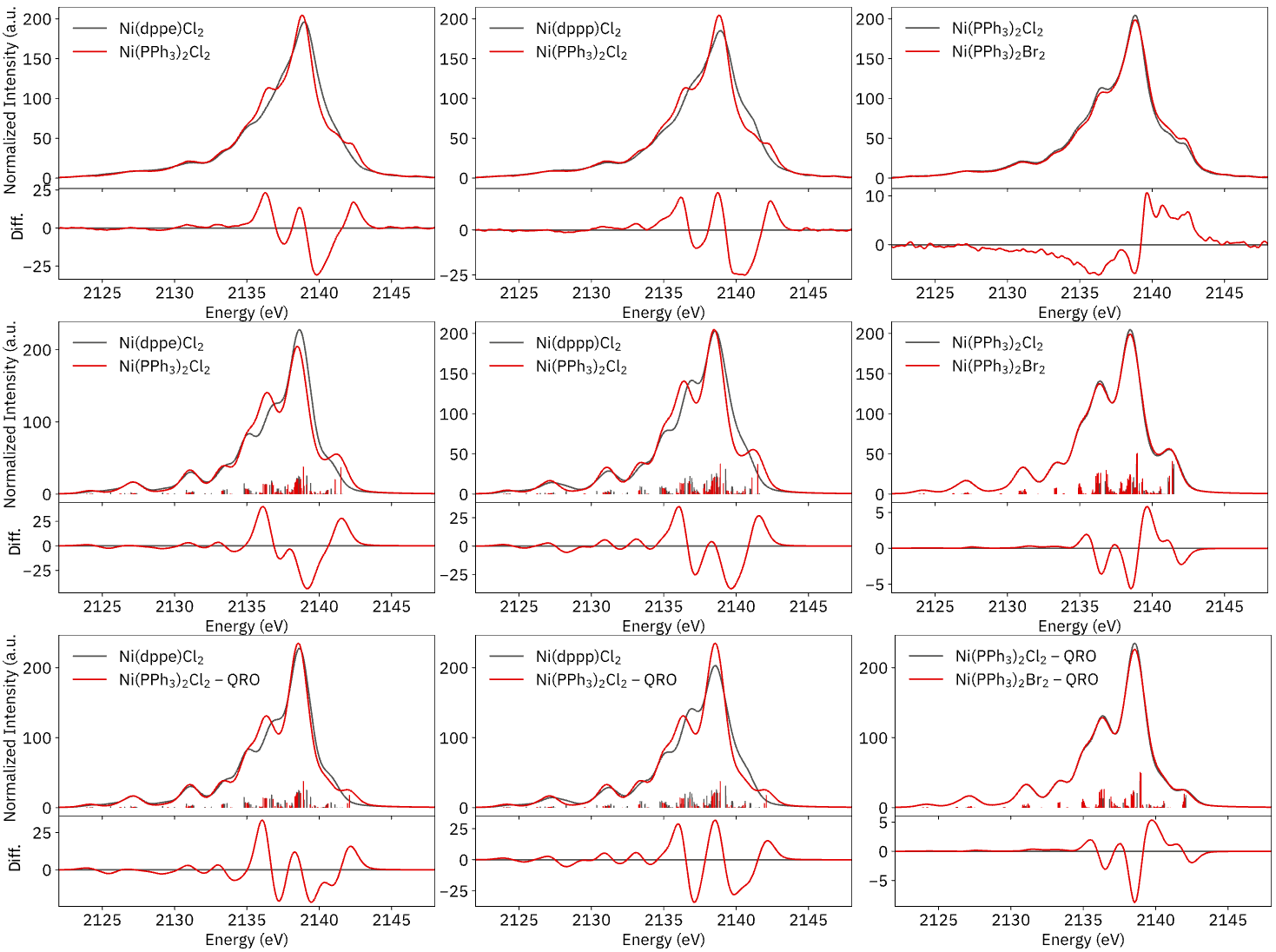


Figure S12. Experimental spectra (top) compared with spectra calculated by the GS 1e1o method with (bottom) and without (middle) use of QROs. Difference spectra (red – black) are shown below each set of spectra.

In the main text, a relatively low Gaussian broadening was chosen for the calculation of Voigt peaks from DFT transitions in order to clearly convey the different spectra features corresponding to groupings of calculated transitions (Figure S13). The overall experimental spectral envelope could be more closely approximated by using a much larger Gaussian broadening; however, a large broadening would obscure features of difference spectra that are present in both the calculated and experimental spectra, such as the difference features at 2139 eV for Ni(dppp)Cl_2_ vs Ni(PPh_3_)_2_Cl_2_ (Figure S13) or at 2141 eV for Ni(PPh_3_)_2_Cl_2_ vs Ni(PPh_3_)_2_Br_2_ (Figure S14).

The fact that an excessive Gaussian broadening better approximates the overall spectral envelope indicates possible shortcomings of the calculation method. For example, there may have been some heterogeneity of P sites in the samples not accounted for in the calculations from single structures, or there could be processes beyond our approximation of one-electron-one-photon transitions to a single core hole.


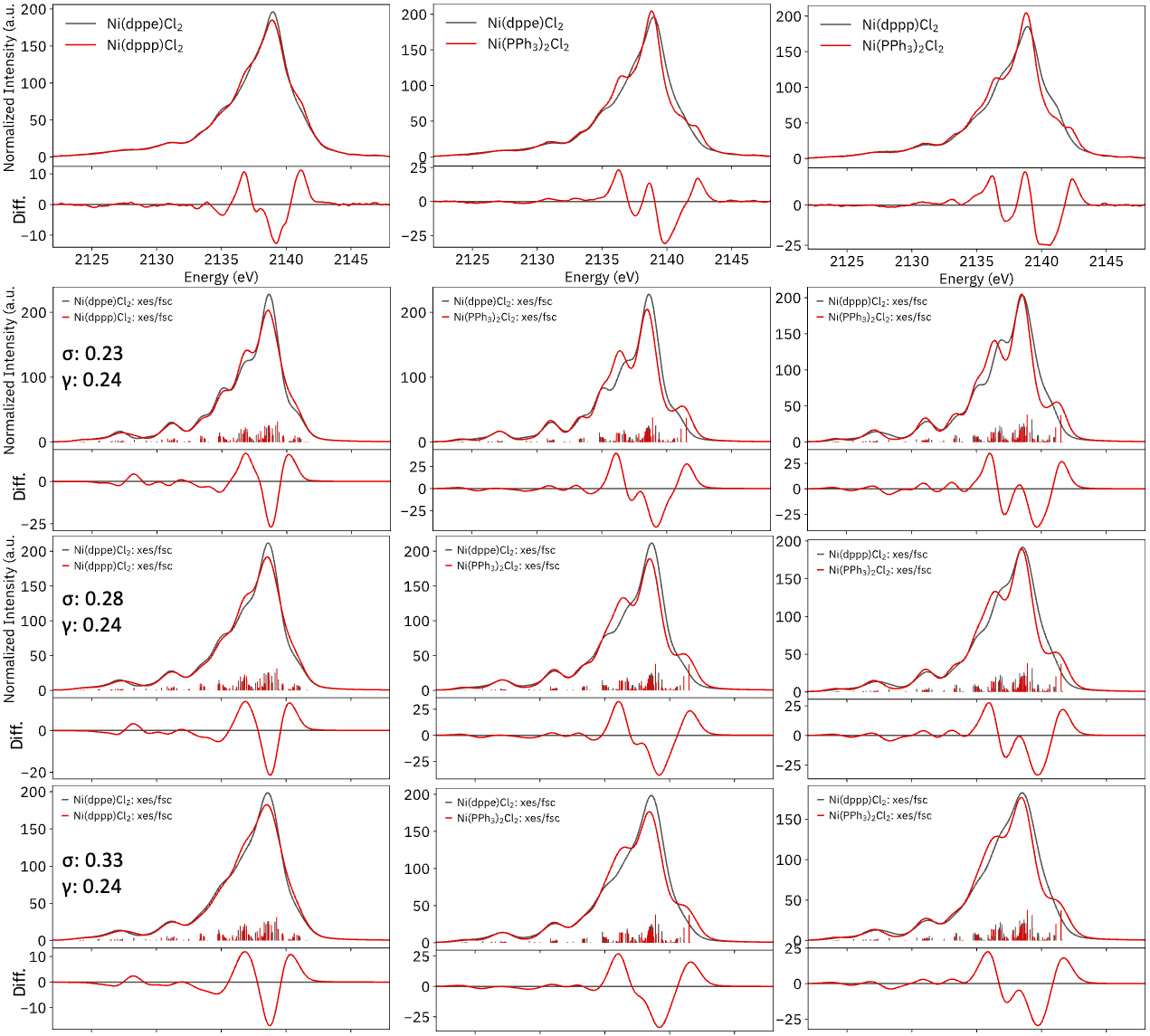


Figure S13. Experimental spectra (top) compared to spectra calculated with the same Lorentzian broadening (γ) and different Gaussian broadenings (σ), with differences (red – black) below, for Ni(dppe)Cl_2_, Ni(dppp)Cl_2_, and Ni(PPh_3_)_2_Cl_2_.


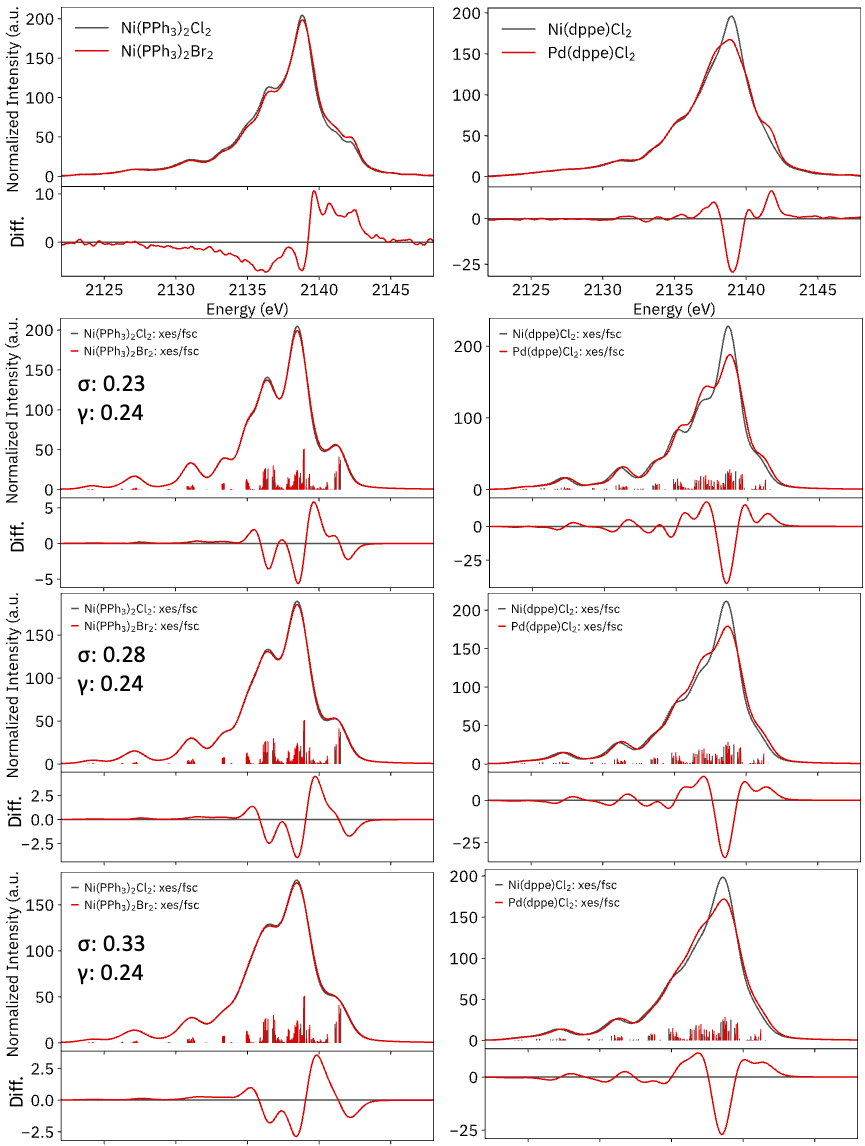


Figure S14. Experimental spectra (top) compared to spectra calculated with the same Lorentzian broadening (γ) and different Gaussian broadenings (σ), with differences (red – black) below, for Ni(dppe)Cl_2_, Pd(dppe)Cl_2_, Ni(PPh_3_)_2_Cl_2_, and Ni(PPh_3_)_2_Br_2_.

## Ni(dppe)Cl_2_ and Ni(dppp)Cl_2_

Ni(dppe)Cl_2_ and Ni(dppp)Cl_2_The spectral differences between Ni(dppe)Cl_2_ and Ni(dppp)Cl_2_ may result from both the length of the electron-donating alkyl linker itself (alkyl effects), and from differing orbital interactions with the [NiCl_2_] moiety resulting from the larger bite angle and orientations of the phenyl groups (chelation effects). Compared to that of Ni(dppe)Cl_2_, the structure of Ni(dppp)Cl_2_ has a larger bite angle (+3.8°), and the bisphosphine is oriented with one P-C(Ph) bond closer to parallel with the Ni coordination plane, and one closer to perpendicular. Similar differences are found in both the crystallographic and optimized structures (Figure S15).

These two effects are not entirely independent or separable conceptually, but a general division might offer some principles for understanding P VtC spectra. Thus, five pairs of models were constructed that emphasize either the alkyl or chelation effect (Figure S16). To investigate the chelation effect, the phosphine alkyl linkers were replaced by a single hydride or methyl group, and the methyl-substituted structures were re-optimized, with Ni–P,Cl distances and all Ni and P angles constrained, to avoid steric clash between the methyls. To investigate the alkyl effect, spectra were calculated with the free ligand frozen in the coordination geometry, the free ligand re-optimized starting from that geometry, and for optimized free PPh_2_(CH_2_)_n=1,2_CH_3_ ligands.


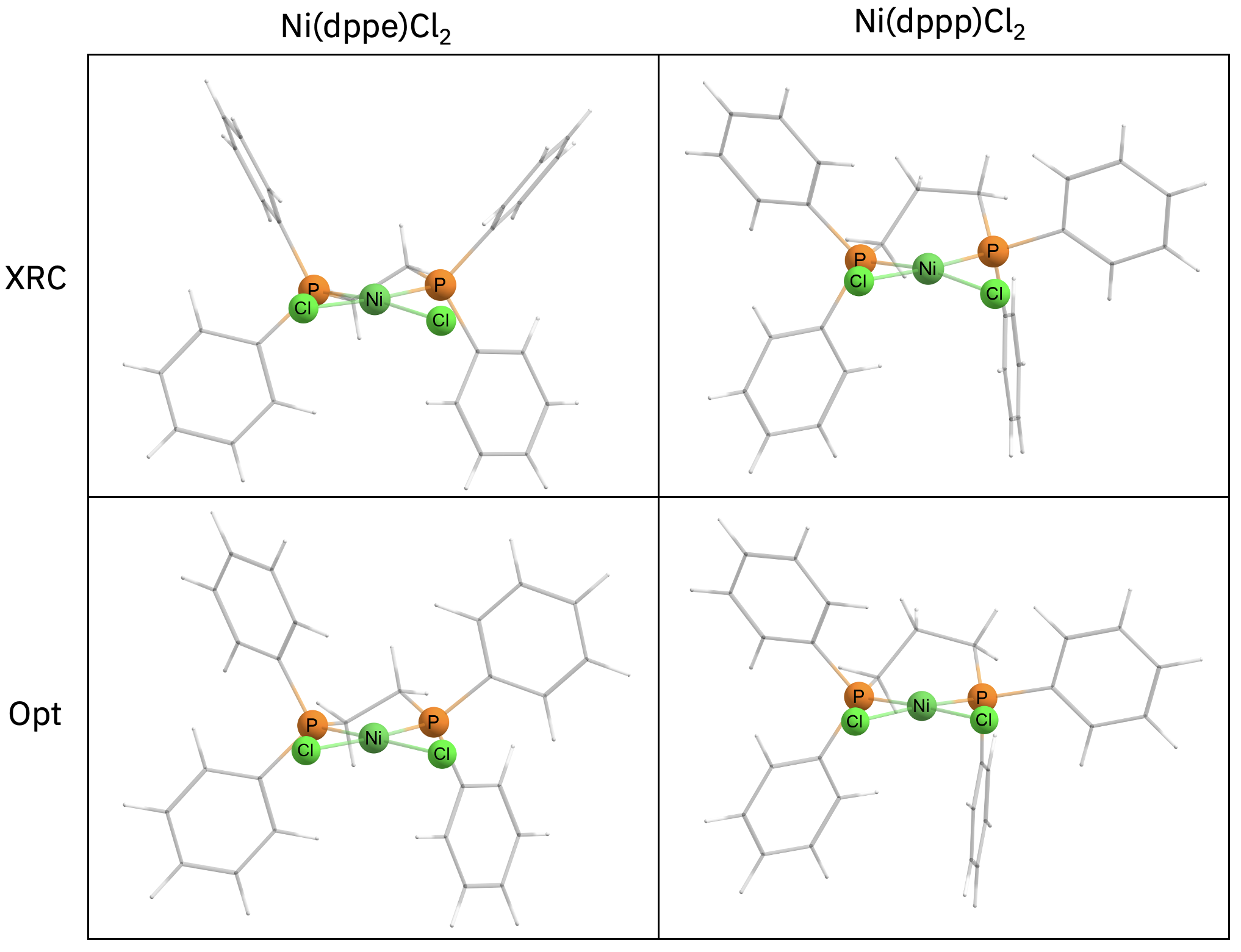


Figure S15. Structures of Ni(dppe)Cl_2_ and Ni(dppp)Cl_2_, including both crystallographic^[45,46]^ (XRC) and fully optimized (Opt) coordinates.


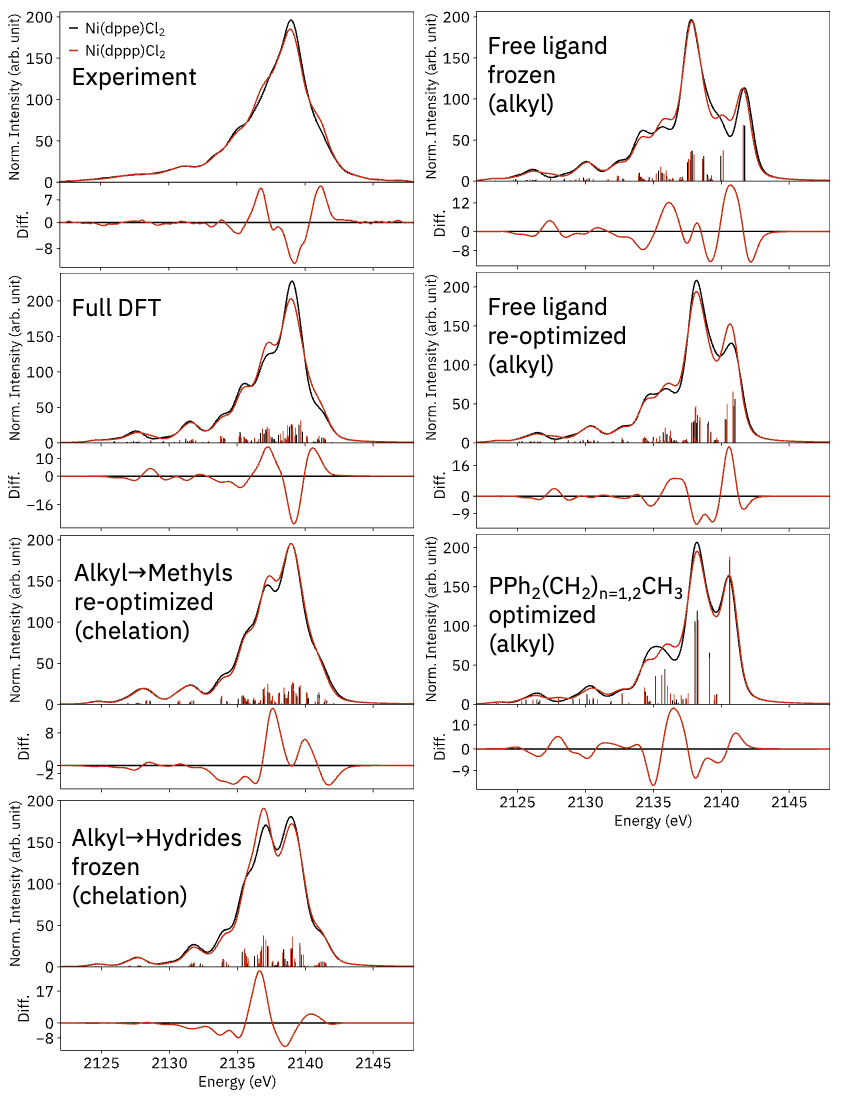


Figure S16. Experimental and calculated P VtC XES of Ni(dppe)Cl_2_ and Ni(dppp)Cl_2_ with differences (dppp – dppe). Calculations are shown for a variety of structural pairs that mostly probe either the electron donation ability of the alkyl linkers or the chelation geometry (bite angle) and orbital interactions with Ni.

## Chloride Substitution

In the main text, the experimental differentiability of Ni(PPh_3_)_2_Cl_2_ and Ni(PPh_3_)_2_Br_2_ by P VtC XES was demonstrated, and the differences were reproduced by DFT calculations. This result shows that P VtC XES can be sensitive to quite subtle changes in metal coordination, an important factor motivating the use of phosphine spectator ligands as an X-ray probe.

The magnitude of changes expected for a single ligand substitution of more catalytic relevance is demonstrated by substituting one chloride in Ni(dppe)Cl_2_ for a methyl or phenyl group (Figure S17).^[47]^ Both substitutions result in changes of comparable magnitude to the P VtC XES. The majority of difference derives from the phosphorus site *trans* to the substitution, including the spectral shift en bloc to lower energy resulting from destabilization of the *trans* P 1*s* orbital by +0.58 eV, and the *cis* P 1*s* orbital by +0.26 eV, compared to the average P 1*s* energy of Ni(dppe)Cl_2_.


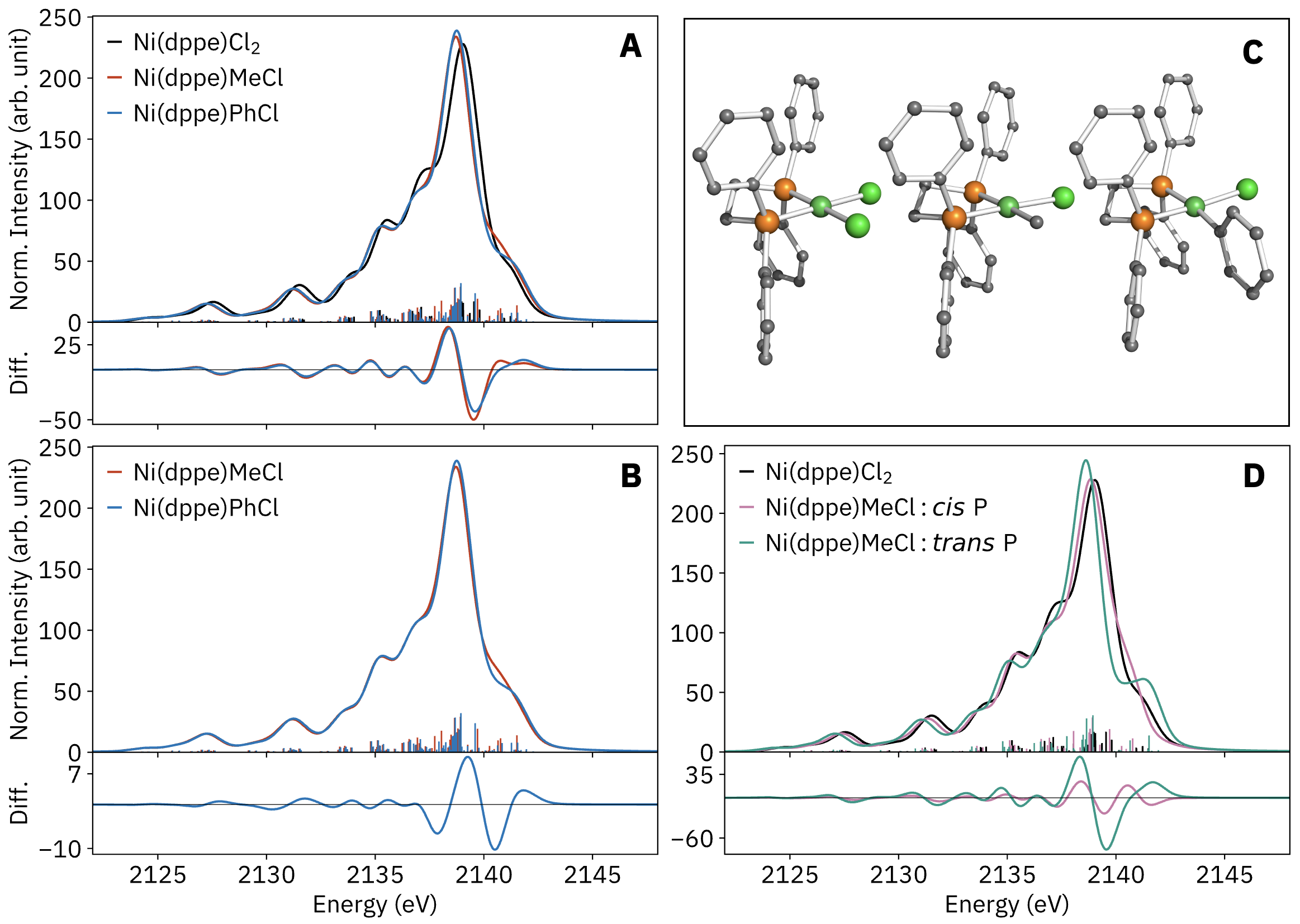


Figure S17. Calculations investigating the effect of substitution of one chloride of Ni(dppe)Cl_2_ for a methyl or phenyl ligand, including A) all three spectra, with differences (methyl-chloro – dichloro in red, phenyl-chloro – dichloro in blue); B) comparison of only Ni(dppe)MeCl and Ni(dppe)PhCl, with difference below (phenylchloride – methylchloride); C) optimized structures of Ni(dppe)Cl_2_, Ni(dppe)MeCl, and Ni(dppe)PhCl; and D) comparison of the total spectrum of Ni(dppe)Cl_2_ (divided by 2) with the individual subsite spectra of Ni(dppe)MeCl, showing larger variation for the P site *trans* to the methyl ligand.

ORCA Input for TD-DFT

As far as we are aware, the use TD-DFT for VtC XES using ORCA has not been previously documented or reported, although it is an established technique.^[28,29]^ The general strategy is to manually swap (rotate) the core orbital and the HOMO, reduce the electron count by one and correct the multiplicity accordingly, and then perform a regular “absorption” TD-DFT calculation with the new HOMO (actually a core orbital) as the only acceptor orbital. This procedure is a special case of the general “core-valence separation” TD-DFT method and results in negative energies and oscillator strengths.^[28]^ See the online Edmond data repository associated with this publication (linked in the main text) for example ORCA input files.

# References

[1] G. Garton, D. E. Henn, H. M. Powell, L. M. Venanzi, *J. Chem. Soc.* **1963**, 3625–3629.

[2] H. J. Bruins Slot, W. K. L. Van Havere, J. H. Noordik, P. T. Beurskens, P. Royo, *Journal of Crystallographic and Spectroscopic Research* **1984**, *14*, 623–627.

[3] B. Corain, B. Longato, R. Angeletti, G. Valle, *Inorganica Chimica Acta* **1985**, *104*, 15–18.

[4] L. Brammer, E. D. Stevens, *Acta Cryst C* **1989**, *45*, 400–403.

[5] A. S. Batsanov, J. a. K. Howard, *Acta Cryst E* **2001**, *57*, m308–m309.

[6] Z. Mathe, O. M. Stepanic, S. Peredkov, S. DeBeer, *Chem. Sci.* **2021**, *12*, 7888–7901.

[7] C. Römelt, S. Peredkov, F. Neese, M. Roemelt, S. DeBeer, *Phys. Chem. Chem. Phys.* **2024**, DOI 10.1039/D4CP00967C.

[8] S. Peredkov, N. Pereira, D. Grötzsch, S. Hendel, D. Wallacher, S. DeBeer, *J Synchrotron Rad* **2024**, *31*, DOI 10.1107/S1600577524002200.

[9] M. Petric, R. Bohinc, K. Bučar, M. Žitnik, J. Szlachetko, M. Kavčič, *Anal. Chem.* **2015**, *87*, 5632–5639.

[10] J. L. Campbell, T. Papp, *Atomic Data and Nuclear Data Tables* **2001**, *77*, 1–56.

[11] P. H. C. Eilers, *Anal. Chem.* **2003**, *75*, 3631–3636.

[12] A. Bowell, *Whittaker-Eilers v0.2.0, Program for Whittaker-Eilers Smoothing and Interpolation*, **2024**.

[13] F. Neese, *WIREs Computational Molecular Science* **2022**, e1606.

[14] J. Tao, J. P. Perdew, V. N. Staroverov, G. E. Scuseria, *Phys. Rev. Lett.* **2003**, *91*, 146401.

[15] S. Grimme, J. Antony, S. Ehrlich, H. Krieg, *Journal of Chemical Physics* **2010**, *132*, DOI 10.1063/1.3382344.

[16] S. Grimme, S. Ehrlich, L. Goerigk, *Journal of computational chemistry* **2011**, *32*, 1545–1614.

[17] V. Barone, M. Cossi, *Journal of Physical Chemistry A* **1998**, *102*, 1995–2001.

[18] M. Garcia-Ratés, F. Neese, *Journal of Computational Chemistry* **2020**, *41*, 922–939.

[19] P. Pollak, F. Weigend, *J. Chem. Theory Comput.* **2017**, *13*, 3696–3705.

[20] T. Lu, F. Chen, *J. Comput. Chem* **2012**, *33*, 580–592.

[21] T. Lu, *The Journal of Chemical Physics* **2024**, *161*, 082503.

[22] C. Adamo, V. Barone, *The Journal of Chemical Physics* **1999**, *110*, 6158–6170.

[23] J. W. Furness, A. D. Kaplan, J. Ning, J. P. Perdew, J. Sun, *J. Phys. Chem. Lett.* **2020**, *11*, 8208–8215.

[24] F. Neese, *J. Am. Chem. Soc.* **2006**, *128*, 10213–10222.

[25] N. Lee, T. Petrenko, U. Bergmann, F. Neese, S. DeBeer, *J. Am. Chem. Soc.* **2010**, *132*, 9715–9727.

[26] C. J. Pollock, S. DeBeer, *Accounts of Chemical Research* **2015**, *48*, 2967–2975.

[27] S. Hugenbruch, H. S. Shafaat, T. Krämer, M. U. Delgado-Jaime, K. Weber, F. Neese, W. Lubitz, S. Debeer, *Physical Chemistry Chemical Physics* **2016**, *18*, 10688–10699.

[28] Y. Zhang, S. Mukamel, M. Khalil, N. Govind, *J. Chem. Theory Comput.* **2015**, *11*, 5804–5809.

[29] D. R. Nascimento, N. Govind, *Physical Chemistry Chemical Physics* **2022**, *24*, 14680–14691.

[30] A. T. B. Gilbert, N. A. Besley, P. M. W. Gill, *J. Phys. Chem. A* **2008**, *112*, 13164–13171.

[31] D. Hait, M. Head-Gordon, *J. Phys. Chem. Lett.* **2021**, *12*, 4517–4529.

[32] M. W. D. Hanson-Heine, M. W. George, N. A. Besley, *The Journal of Chemical Physics* **2017**, *146*, 094106.

[33] S. Tetef, V. Kashyap, W. M. Holden, A. Velian, N. Govind, G. T. Seidler, *J. Phys. Chem. A* **2022**, *126*, 4862–4872.

[34] K. Saini, A. N. Nair, A. Yadav, L. G. Enriquez, C. J. Pollock, S. D. House, S. Yang, X. Guo, S. T. Sreenivasan, *Advanced Energy Materials* **2023**, *13*, 2302170.

[35] H. Lim, M. L. Baker, R. E. Cowley, S. Kim, M. Bhadra, M. A. Siegler, T. Kroll, D. Sokaras, T.-C. Weng, D. R. Biswas, D. M. Dooley, K. D. Karlin, B. Hedman, K. O. Hodgson, E. I. Solomon, *Inorg. Chem.* **2020**, *59*, 16567–16581.

[36] S. DeBeer George, T. Petrenko, F. Neese, *Inorganica Chimica Acta* **2008**, *361*, 965–972.

[37] L. K. Sørensen, E. Kieri, S. Srivastav, M. Lundberg, R. Lindh, *Phys. Rev. A* **2019**, *99*, 013419.

[38] N. O. Foglia, D. Maganas, F. Neese, *J. Chem. Phys.* **2022**, *157*, 084120.

[39] B. L. Geoghegan, Y. Liu, S. Peredkov, S. Dechert, F. Meyer, S. DeBeer, G. E. Cutsail, *J. Am. Chem. Soc.* **2022**, *144*, 2520–2534.

[40] O. McCubbin Stepanic, J. Ward, J. E. Penner-Hahn, A. Deb, U. Bergmann, S. DeBeer, *Inorg. Chem.* **2020**, *59*, 13551–13560.

[41] Q. Yang, Y. Deng, H. Yang, H. Zhao, P. Yao, J. Chen, Z. Ma, B. Fan, *ACS Catal.* **2025**, *15*, 2666–2676.

[42] S. Seo, T. Shin, I. Choi, H. Kim, *ACS Catal.* **2024**, *14*, 11113–11120.

[43] R. L. Harlow, R. J. McKinney, J. F. Whitney, *Organometallics* **1983**, *2*, 1839–1842.

[44] Z. Mathe, D. A. Pantazis, H. B. Lee, R. Gnewkow, B. E. Van Kuiken, T. Agapie, S. DeBeer, *Inorg. Chem.* **2019**, *58*, 16292–16301.

[45] S. C. Davies, R. A. Henderson, D. L. Hughes, K. E. Oglieve, *J. Chem. Soc., Dalton Trans.* **1998**, 425–432.

[46] M. J. O’Neill, T. Riesebeck, J. Cornella, *Angewandte Chemie International Edition* **2018**, *57*, 9103–9107.

[47] T. Yamamoto, T. Kohara, K. Osakada, A. Yamamoto, *Bulletin of the Chemical Society of Japan* **1983**, *56*, 2147–2153.
